# Therapeutic Interaction Features of AI Chatbots in Depression Interventions: Systematic Review and Meta-Analysis

**DOI:** 10.2196/88697

**Published:** 2026-06-30

**Authors:** Ting Huang, Shuangyu Li, Yanzhong Wang, Wei Liu

**Affiliations:** 1 Department of Engineering, King's College London, S2.20, Strand Building, Strand Campus, Strand, London, WC2R 2LS, United Kingdom, 44 20 7836 5454; 2 Department of Interdisciplinary Humanities, Faculty of Arts and Humanities, King's College London, London, United Kingdom; 3 Department of Population Health Sciences, School of Life Course and Population Sciences, Faculty of Life Sciences & Medicine, King's College London, London, United Kingdom

**Keywords:** depression, AI-driven chatbot, user adherence, digital mental health intervention, interaction design, meta-analysis

## Abstract

**Background:**

Depression is a prevalent mental health disorder and a leading cause of disability worldwide, creating substantial personal and societal burdens. Digital mental health interventions have emerged as accessible and scalable solutions, with artificial intelligence (AI)–driven chatbots increasingly applied to deliver therapeutic content, monitor symptoms, and provide personalized support. However, limited evidence exists on how chatbot interaction features influence treatment adherence and clinical outcomes in depression.

**Objective:**

This systematic review aimed to evaluate the clinical effectiveness of AI-driven chatbots for depression and to examine the associations between chatbot characteristics, treatment outcomes, and user adherence.

**Methods:**

A systematic review and meta-analysis were conducted following PRISMA (Preferred Reporting Items for Systematic Reviews and Meta-Analyses) 2020 guidelines, searching 6 databases (Web of Science, Scopus, PubMed, IEEE Xplore, Embase, and APA PsycINFO) for randomized controlled trials (RCTs) published before May 30, 2025. Eligible studies involved individuals with depression or depressive symptoms receiving AI-driven chatbot, conversational agent, or virtual therapist interventions, with outcomes assessed using the Patient Health Questionnaire-9 (PHQ-9). Data extraction included chatbot type, interaction features, adherence, and standardized mean differences (SMDs) for symptom change. Risk of bias was assessed using the Cochrane Risk of Bias tool version 2 (RoB 2). Random-effects meta-analyses were performed with the Hartung-Knapp-Sidik-Jonkman adjustment. This review was preregistered on the Open Science Framework.

**Results:**

A total of 11 RCTs involving 2220 participants (1091 in the intervention and 1129 in the control groups) were included. Using a random-effects model with Hartung-Knapp-Sidik-Jonkman adjustment, AI-driven chatbots showed a small-to-moderate reduction in depressive symptoms compared with control conditions, but the effect was not statistically significant (SMD=−0.46, 95% CI −1.02 to 0.10; *P*=.01; 95% prediction interval −1.50 to 0.58). Subgroup analyses of adherence did not show significant differences across the reported chatbot-type subgroups. In contrast, exploratory analyses of interaction features revealed more consistent patterns for adherence. Emotional responsiveness, structured feedback strategies, and interaction frequency were associated with higher adherence in high-scoring subgroups, whereas dialogue depth, self-disclosure encouragement, and user agency level showed weaker or inconsistent associations. For clinical outcomes, associations with interaction features were less consistent and more heterogeneous.

**Conclusions:**

This systematic review provides an interaction-focused synthesis of AI-driven chatbot interventions for depression, examining how interaction features relate to clinical outcomes and user adherence. Although overall effects were not statistically significant, emotional responsiveness, structured feedback, and interaction frequency were consistently associated with higher adherence. Engagement and outcomes may be influenced by distinct mechanisms. Limitations include the small number of RCTs, heterogeneity, reliance on study-reported descriptions, and potential publication bias. These findings highlight the importance of interaction design in developing scalable digital mental health interventions.

## Introduction

Depression is one of the leading causes of disability worldwide. According to the World Health Organization (WHO), more than 280 million people worldwide are affected, with a prevalence of 3.8% in the general population and 5.7% among adults aged 60 years or older [1]. Depression contributes substantially to the global burden of disease and has serious effects on quality of life, productivity, and physical health [2]. Although face-to-face psychotherapy and pharmacological treatments are effective, access remains limited due to workforce shortages, stigma, and geographical barriers [3,4].

To address these barriers, digital mental health interventions (DMHIs) have rapidly emerged as scalable, accessible alternatives [5]. For example, internet-based cognitive behavioral therapy (iCBT) allows remote treatment, while mobile apps support mood tracking and self-management [6]. Artificial intelligence (AI)–driven chatbots, which have attracted growing attention in this field, offer continuous support and characterize sustained, language-based interaction.

Early DMHIs relied on web-based psychoeducation and structured cognitive behavioral therapy programs with fixed content and limited interactivity [7,8]. With the expansion of mobile technologies, these interventions shifted toward app-based formats, providing more flexible, on-demand support through features such as reminders, mood tracking, and self-help tools [9]. However, these features largely remained task-based and relied on predefined responses rather than sustained, context-sensitive interaction.

A meta-analysis by Lattie et al [10], examined a wide range of DMHIs, including iCBT, app-based interventions, messaging systems, and virtual reality platforms. The findings showed that DMHIs can achieve clinical outcomes comparable to traditional therapies for mild to moderate depression [10]. However, the review provided limited differentiation between interaction types and did not examine their specific roles in shaping clinical outcomes. This suggests that interaction has often been treated as a secondary feature rather than a core mechanism in DMHIs.

Despite advances, DMHIs still face high dropout rates and low retention. This persistent issue is known as the “Law of Attrition,” which highlights the challenge of maintaining long-term engagement in digital interventions [11]. Recent work suggests evaluating both engagement and clinical effectiveness rather than focusing on a single metric [12]. These concerns are especially relevant for chatbot-based interventions, which rely on ongoing conversational interaction for continued use.

Existing studies show that traditional DMHIs, such as web-based psychoeducation and iCBT, struggle with user retention and lack personalized, interactive support. These shortcomings underscore the importance of interaction for improving therapeutic effectiveness and adherence. In response, computerized cognitive behavioral therapy emerged in the late 20th century. Since the mid-2010s, AI has been integrated into health care, leading to the rise of AI-driven chatbots [13].

Unlike earlier DMHIs, AI chatbots are fundamentally interaction-centered, with therapeutic support delivered primarily through ongoing conversational exchange. Consistent with prior definitions of conversational agents in digital health, they are distinguished from nonconversational digital interventions by their capacity for sustained, multiturn dialogue, which forms the core therapeutic mechanism [14]. A meta-analysis by Li et al [15] found that AI-based conversational agents significantly reduced depressive symptoms and further suggested that user experience depends on factors such as therapeutic alliance with AI, content engagement, and communication quality. This points to the need to examine the mechanisms through which chatbot interactions produce clinical benefit. However, the role of specific interaction features in shaping these mechanisms remains insufficiently examined.

By leveraging natural language processing (NLP) and machine learning (ML), chatbots can simulate human conversation and provide round-the-clock access to psychoeducation, cognitive behavioral therapy exercises, and emotional support [16,17]. Recent studies have shown that chatbots such as Woebot (Woebot Health, Inc) and Wysa (Wysa Health) can reduce depressive symptoms across diverse populations [18,19]. At the same time, more recent work suggests that integrating large language models (LLMs) into mental health care remains at an early stage. A UK-based evaluation involving 132 participants found that although many users were familiar with systems such as ChatGPT (OpenAI) and Doubao (ByteDance), their clinical use in mental health care was still limited [20]. Similarly, semistructured interviews with German adolescents experiencing depressive symptoms showed that participants generally held cautiously positive attitudes toward chatbots, while also expressing diverse and sometimes conflicting expectations regarding personalization [21]. Together, these findings suggest that LLMs may extend chatbot capabilities in contextual awareness, empathy, and personalized interaction, but their clinical role is still evolving [22].

Recent research has highlighted the importance of aligning chatbot design with user preferences and needs in mental health care. For instance, Kim et al [23] used a mixed logit model to analyze user choice data, showing that preferences for mental health chatbots are broadly consistent with those observed in traditional counseling contexts. They therefore emphasized the importance of human-centered design in the development of health care chatbots. Personalization is also critical across different user groups. For example, chatbots designed for older adults should incorporate age-friendly interfaces that account for age-related physiological characteristics [24]. In addition, user personality traits and usage contexts have been identified as important factors in shaping chatbot interaction. Furini et al [25], based on data from multiple user profiles and scenarios, highlighted the need to integrate personality and health conditions into chatbot interactions to improve engagement and outcomes. Together, these studies underscore the importance of interaction design in mental health chatbots. However, their findings remain fragmented and difficult to synthesize across different interaction features and user groups.

Trust is a key factor in mental health care, as therapeutic effectiveness depends on the development of rapport, empathy, and credibility between clinician and patient. In traditional therapy, clinicians build trust through empathic listening, contingent feedback, and adaptive communication. Translating these mechanisms into chatbot interactions remains challenging. Dong and Wu [26] explored how the perceived status of a health care chatbot influences patient trust. Their findings suggest that when chatbots assume a high-status role and provide contextually contingent responses, users report lower anxiety when interacting with AI systems. However, how specific interaction features such as empathy expression or feedback strategies can be adaptively adjusted to promote trust remains insufficiently studied [27-29]. This is particularly relevant given the skepticism that both patients and clinicians often express toward AI in health care, which may compromise trust-building and acceptance. Existing studies suggest that embedding personalization mechanisms into interactional features could strengthen user trust, thereby improving adherence and clinical outcomes [30-32]. Overall, trust represents a key interactional mechanism in mental health chatbots. However, the effects of specific interaction features on trust, adherence, and clinical outcomes have not been systematically examined.

This understanding of how AI-driven chatbots support depression care remains fragmented. Existing research has largely focused on overall clinical effectiveness, while systematic examination of how specific interaction features (eg, dialogue depth, feedback strategies, and emotional responsiveness) and content types (eg, self-disclosure prompts and goal-setting) relate to therapeutic outcomes remains limited. Moreover, evidence on how these interactional characteristics influence user adherence is sparse and often indirect. Addressing these gaps is essential to inform the design of chatbot-based interventions that not only reduce depressive symptoms but also sustain engagement and trust over time. Against this background, the present study synthesizes existing evidence to examine how interaction and content features of AI-driven chatbots relate to clinical effectiveness and user adherence in depression care.

This systematic review therefore aims to address the following research questions (RQs):

1. RQ1: What is the overall clinical effectiveness of AI-driven chatbots in depression interventions?
2. RQ2: How does user adherence vary across AI-driven chatbots based on different AI technologies?
3. RQ3: How do the interaction features of AI-driven chatbots influence both treatment outcomes and user adherence?

## Methods

### Search Strategy

This systematic literature search was conducted to identify research on AI-driven chatbot interventions for the treatment of depression. The objective was to evaluate the influence of chatbot-based digital tools on clinical outcomes, including symptom improvement, treatment effectiveness, and user adherence. Studies were included only if chatbots were used as therapeutic tools, while those focusing exclusively on diagnosis, screening, or prediction were excluded.

The search was conducted across 6 major academic databases, including Scopus, Web of Science, PubMed, IEEE Xplore, APA PsycINFO (Ovid), and Embase (Ovid). The strategy incorporated 2 primary concept domains, namely depression and chatbot or conversational systems. Only peer-reviewed journal articles and conference proceedings published in English, with coverage up to May 30, 2025, were included, as detailed in Textbox 1.

The search strategy was developed in accordance with guidance from the Cochrane Handbook. Consistent conceptual blocks were applied across all databases, with syntax adapted for each platform. Full database-specific search strategies are provided in Multimedia Appendix 1. Reporting of the search strategy and process adhered to PRISMA-S (Preferred Reporting Items for Systematic Reviews and Meta-Analyses Literature Search Extension) guidelines to ensure transparency and reproducibility [33]. The PRISMA-S checklist is presented in Checklist 1.

Textbox 1. **Search strategy.**

**Search topic**

1. Depression and depressive disorders (eg, “depression,” “major depressive disorder,” “major depressive disorder,” “dysthymia”)
2. Chatbot and conversational agent systems (eg, “chatbot,” “conversational agent,” “virtual agent,” “dialogue system,” “Woebot,” “Wysa”)
3. Intervention and treatment-related terms (eg, “intervention,” “therapy,” “treatment,” “counselling,” “psychotherapy,” “cognitive behavioral therapy,” “CBT,” “digital mental health,” “digital therapy,” “psychological intervention”)

**Search example**

TS=((depress* OR “major depressive disorder” OR “MDD” OR dysthymi* OR “persistent depressive disorder” OR “depressive disorder*” OR “depressive symptom*” OR “depressive episode*” OR “recurrent depressive disorder” OR “unipolar depression” OR “mood disorder*” OR “affective disorder*” OR “subclinical depression” OR “subthreshold depression”)

AND

(chatbot* OR “chat bot*” OR “conversational agent*” OR “conversational AI” OR “dialogue system*” OR “dialog system*” OR “virtual therapist*” OR “virtual agent*” OR “relational agent*” OR “embodied agent*” OR “mental health bot*” OR Woebot OR Wysa OR Tess OR Youper OR Replika OR Ellie)

AND

(intervention* OR therap* OR treatment* OR counsel* OR psychotherap* OR “cognitive behavioral therapy” OR CBT OR “digital mental health” OR “digital therap*” OR “psychological intervention*”))

### Selection Criteria

#### Inclusion Criteria

Studies were included if they met all of the following criteria:

1. Population: participants were individuals experiencing depression or related affective conditions, including major depressive disorder, dysthymia, clinical depression, or comorbid anxiety symptoms.
2. Intervention: the study examined a digital intervention in which an AI-driven chatbot, conversational agent, or virtual therapist played a central role in delivering therapeutic content (eg, psychoeducation, cognitive behavioral therapy, counseling support, or mood regulation exercises).
3. Purpose of intervention: the chatbot was used with the explicit aim of reducing depressive symptoms, improving psychological well-being, or supporting behavioral change. Both standalone and blended interventions (chatbot plus human support) were eligible.
4. Outcome: the study reported at least one outcome related to treatment effectiveness, symptom improvement, or adherence.
5. Study type: randomized controlled trials (RCTs).
6. Publication status and language: full-text available in English; published as a journal article or conference proceeding.

#### Exclusion Criteria

Studies were excluded if they met any of the following:

1. Nontherapeutic use: the chatbot was used solely for screening, diagnosis, symptom monitoring, or predictive modeling without a therapeutic component.
2. Non-AI or rule-based systems: the system used was not AI-driven (static decision-tree chatbots without learning capacity).
3. Nondepressive focus: the intervention targeted conditions unrelated to depression, such as bipolar disorder, schizophrenia, psychosis, dementia, or autism spectrum disorder.
4. Theoretical or technical papers: studies describing only the design, technical architecture, or conceptual framework of a chatbot without user evaluation or outcome reporting.
5. Lack of baseline data: studies that did not report baseline outcome measures for depression.
6. Inconsistent outcome measurement: pre-post comparisons not based on validated depression scales, specifically the Patient Health Questionnaire-9 (PHQ-9).
7. Gray literature: Editorials, protocols, opinion pieces, preprints, or dissertations.

### Data Extraction

All records identified from the 6 databases, including Scopus, Web of Science, PubMed, IEEE Xplore, APA PsycINFO (Ovid), and Embase (Ovid), were imported into EndNote (version 21; Clarivate) for management and initial filtering based on 11 predefined bibliographic fields, including author, year, abstract, and keywords. A total of 3372 records were retrieved. After removing 4 erroneous records, 3368 records were retained for deduplication, resulting in 2097 unique articles. Titles and abstracts were independently screened by 2 reviewers (TH and WL) against the eligibility criteria. Any discrepancies were resolved through discussion with a third reviewer. In total, 87 articles were selected for full-text assessment, of which 8 met the inclusion criteria. An additional 3 eligible studies were identified through snowballing. Overall, 11 studies were included in the final analysis and subjected to meta-analysis. The screening and selection process is summarized in the PRISMA (Preferred Reporting Items for Systematic Reviews and Meta-Analyses; Checklist 2) flow diagram.

In addition to extracting general study characteristics, 6 interaction features of the chatbots were assessed, including interaction frequency, emotional responsiveness, self-disclosure encouragement, dialogue depth, feedback strategy, and user agency level. Each feature was rated on a 5-point scale (1=very low-5=very high) by 2 independent reviewers (TH and WL). A third reviewer adjudicated when ratings differed by ≥1 point, and the final score for each feature was calculated as the mean of the available ratings. Interrater reliability across all ratings was good (overall intraclass correlation coefficient [ICC]=0.71); detailed results are presented in the Multimedia Appendix 2.

For clarity, the 6 features were defined as follows: interaction frequency (the intensity and regularity of user-chatbot exchanges), emotional responsiveness (the chatbot’s ability to adaptively provide empathetic responses), self-disclosure encouragement (prompts guiding users to share personal experiences or emotions), dialogue depth (the richness and reflectiveness of conversations), feedback strategy (the presence of timely and tailored prompts or evaluative responses), and user agency level (the degree of control and choice available to the user).

User adherence was operationalized as intervention completion, defined as the proportion of participants who completed the intervention protocol relative to the number initially enrolled. This completion-based measure was consistently reported across the included studies and was therefore adopted to enable quantitative synthesis of adherence outcomes.

### Data Quality

The methodological quality of the included studies was assessed independently by 2 reviewers (TH and WL) using the Cochrane Risk of Bias 2 (RoB 2) tool for RCTs. The tool evaluates potential biases across five domains: (1) bias arising from the randomization process, (2) bias due to deviations from intended interventions, (3) bias due to missing outcome data, (4) bias in the measurement of the outcome, and (5) bias in the selection of the reported result. Each domain was rated as “low risk,” “some concerns,” or “high risk” according to the signaling questions provided by RoB 2 guidelines.

Any discrepancies in assessments between the 2 reviewers (TH and WL) were resolved through discussion; if disagreement persisted, a third reviewer was consulted. The final risk-of-bias assessments were summarized in tabular and graphical form.

### Statistical Analysis

Meta-analyses were conducted using random-effects models. Standardized mean differences (SMDs) were calculated for continuous outcomes, and odds ratios (ORs) were calculated for dichotomous outcomes. Heterogeneity was quantified using the *I*^2^ and statistics [34]. Prediction intervals (PIs) were calculated to estimate the range of true effects in future comparable settings [35]. To provide more robust CI estimates, particularly given the relatively small number of included studies, the Hartung-Knapp-Sidik-Jonkman (HKSJ) adjustment was applied [36]. The DerSimonian-Laird estimator was also used for comparison [37]. To assess potential small-study effects, funnel plots were visually inspected. Egger regression test [38], the Begg-Mazumdar rank correlation test [39], and the Duval and Tweedie trim-and-fill procedure were applied where appropriate (≥10). All analyses were conducted in R (version 4.5.1; R Foundation for Statistical Computing).

## Results

### Overview

**Figure 1.**
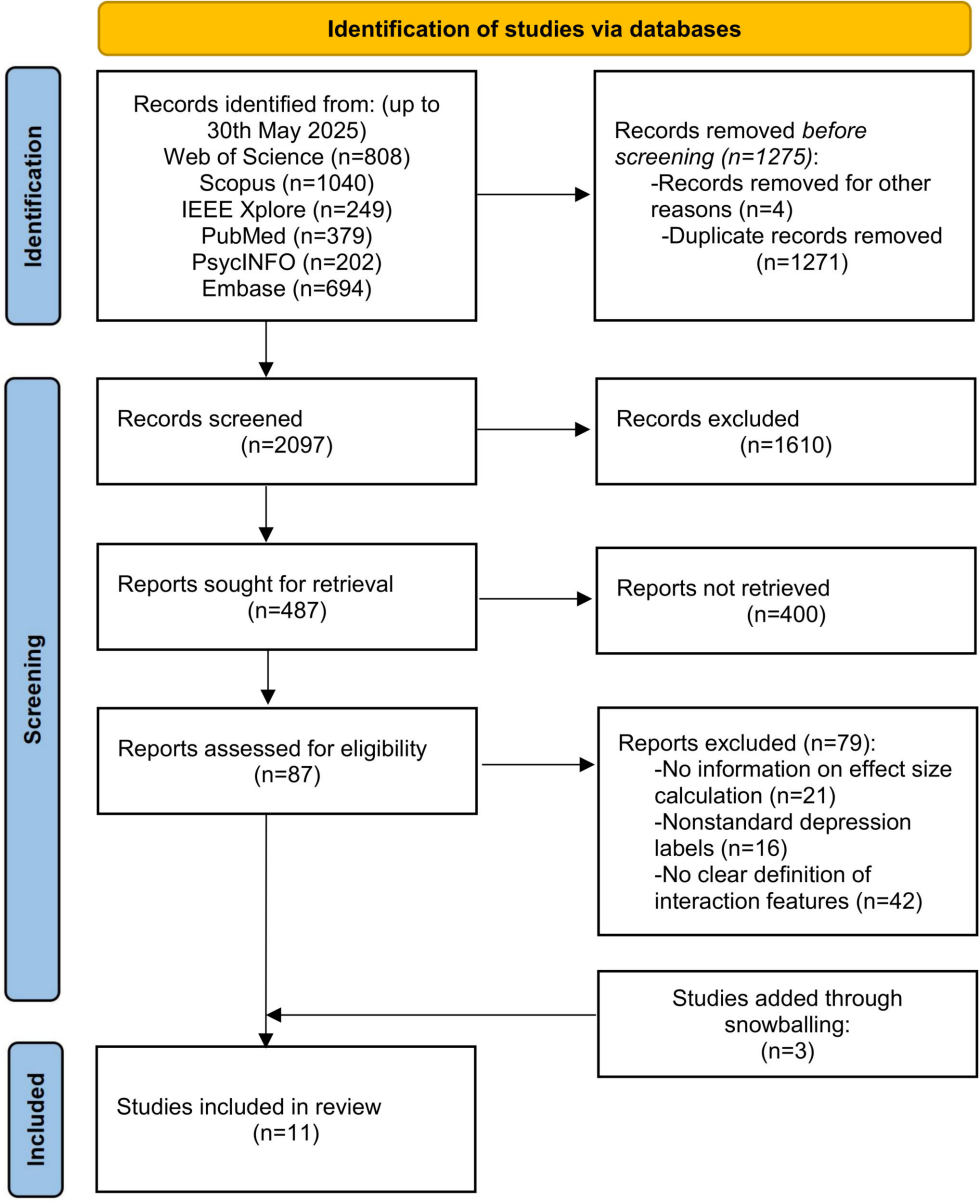
PRISMA (Preferred Reporting Items for Systematic Reviews and Meta-Analyses) flowchart. Study selection for systematic review.

The study selection process is shown in Figure 1. After full-text screening, a total of 11 articles were included in the analysis. In total, 2220 participants (1091 in the intervention and 1129 in the control groups) were included in our analysis. A summary of the characteristics of the studies is presented in Table 1.

**Table 1.** Summary of all studies included in the review.

Item	Study and year	Country	Initial participants (n)		Used for effect size (n)		Age (years), mean (SD) or median (IQR)	Study methodology	Scales	Chatbot name or platform
			Intervention	Control	Intervention	Control				
1	Chen et al (2025) [40]	China	62	41	62	41	—^a^	2-armed RCT^b^ with 2 parallel groups recruited from Hong Kong	PHQ-9^c^ GAD-7^d^	COVID-19 information chatbot (University of Hong Kong)
2	Fitzpatrick et al (2017) [41]	United States	34	36	31	25	22.2 (2.33)	2-armed RCT with 2 groups recruited from a university community social media site	PHQ-9 GAD-7	Woebot (Woebot Health, Inc)
3	He et al (2022) [42]	China	49	49	44	32	18.78 (3.18)	3-arm RCT performed at a university in Tianjin, China	PHQ-9	XiaoE (Tianjin University; technical support from Xiaomi Corporation)
4	Kang and Hong (2024) [43]	South Korea	22	10	15	3	Experimental: 23.5 (1.78) Control: 22.9 (1.85)	2-armed RCT with participants recruited from Sungkyunkwan University’s Colleges of Natural Sciences and Humanities and Social Sciences in Seoul, South Korea	UCLA^e^ PHQ-9	Woebot (Woebot Health, Inc)
5	Karkosz et al (2024) [44]	Poland	40	41	33	35	Experimental: 26.60 (5.06) Control: 24.76 (4.01)	2-armed RCT with participants recruited via Facebook (Meta) and Instagram (Meta) advertisements	CESD-R^f^ PANAS^g^ PHQ-9 PSWQ^h^ R-UCLA^i^ STAI^j^ SWLS^k^	Fido (Szkoła Wyższa Psychologii Społecznej University research team)
6	Liu et al (2022) [45]	China	41	42	33	30	23.08 (1.76)	2-armed RCT with participants recruited from 3 different universities in China	PHQ-9 GAD-7	XiaoNan (South China University of Technology)
7	Sabour et al (2023) [46]	China	90	121	70	105	—	3-arm RCT with participants recruited from social media platforms	PHQ-9 GAD-7 PANAS ISI^l^	ES-Bot^m^ (part of Emohaa, Beijing Lingxin Intelligent Technology Co, Ltd)
8	Tong et al (2024) [47]	China	140	145	118	132	26.45 (8.37)	2-armed RCT with participants recruited from social media platforms	SUPPH^n^ eTAP^o^ SCBI^p^ MHLS^q^ PHQ-9 GAD-7 MAAS^r^ PERMA^s^	Boon (Chinese University of Hong Kong)
9	Ulrich et al (2024) [48]	Switzerland	70	70	42	56	26.7 (6.3)	2-armed RCT with participants recruited from a population of university students in Switzerland	PHQ-9 GAD-7 PHQ-15 HAPA^t^	MISHA (Szkoła Wyższa Psychologii Społecznej University research team)
10	Vereschagin et al (2024) [49]	Canada	743	746	591	619	20 (19-23)	2-armed RCT with participants recruited from the University of British Columbia (UBC) Vancouver campus	GAD-7 PHQ-15 USAUDIT-C^u^	Minder (University of British Columbia)
11	Yasukawa et al (2024) [50]	Japan	74	75	52	51	41.4 (11.1)	2-armed RCT with participants recruited from Japan	PHQ-9 GAD-7 CBT^v^ skills SWLS^w^ WHO-5^x^ WSAS^y^ UWES^z^	EPO/LINE (Sony Group Corporation)

a Not applicable.

b RCT: randomized controlled trial.

c PHQ: Patient Health Questionnaire.

d GAD-7: Generalized Anxiety Disorder 7-item scale.

e UCLA: UCLA Loneliness Scale.

f CESD-R: Center for Epidemiologic Studies Depression Scale Revised.

g PANAS: Positive and Negative Affect Scale.

h PSWQ: Penn State Worry Questionnaire.

i R-UCLA: Revised UCLA Loneliness Scale.

j STAI: State-Trait Anxiety Inventory.

k SWLS: Satisfaction With Life Scale.

l ISI: Insomnia Severity Index.

m ES: emotional support.

n SUPPH: strategies used by people to promote health.

o eTAP: e-Therapy Attitude and Process Questionnaire.

p SCBI: Self-Care Behaviors Inventory.

q MHLS: Mental Health Literacy Scale.

r MAAS: Mindful Attention Awareness Scale.

s PERMA: positive emotion, engagement, relationships, meaning, and accomplishment.

t HAPA: health action process approach.

u USAUDIT-C: US Alcohol Use Disorders Identification Test–Consumption Scale.

v CBT: cognitive behavioral therapy.

w SWLS: Satisfaction with Life Scale.

x WHO-5: World Health Organization-Five Well-Being Index.

y WSAS: Work and Social Adjustment Scale.

z UWES: Utrecht Work Engagement Scale.

### Overall Clinical Effectiveness of AI-Driven Chatbots in Depression Interventions

**Figure 2.**
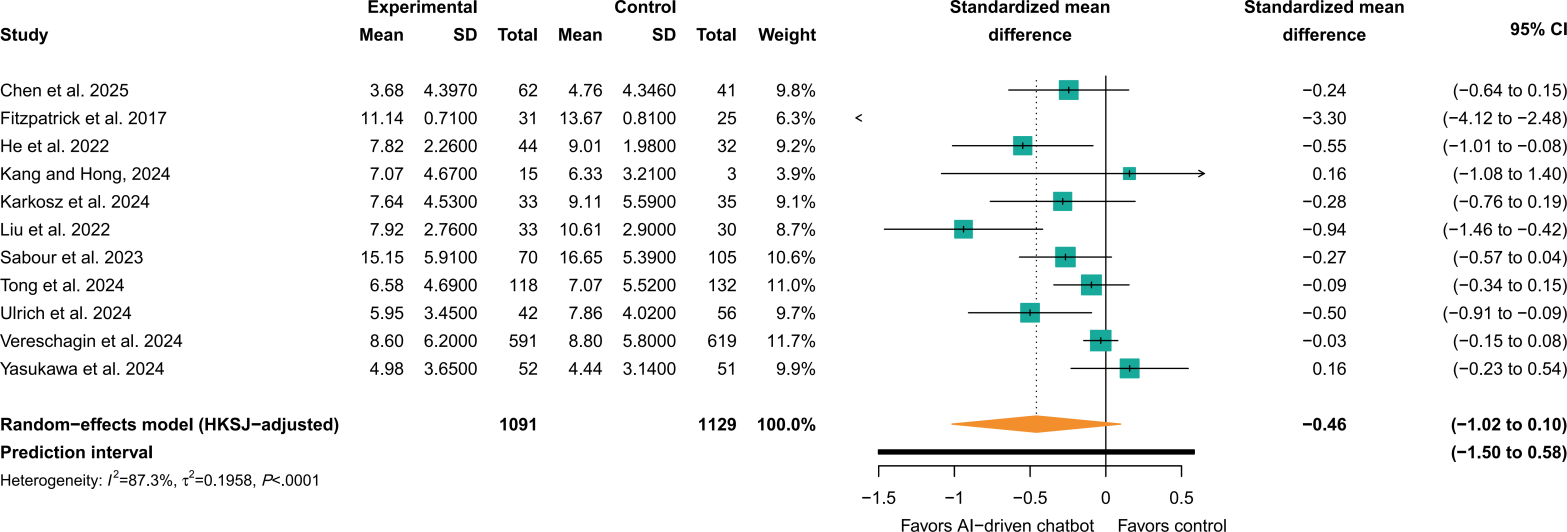
Forest plot of the overall clinical effect of artificial intelligence (AI)–driven chatbots on depressive symptoms. A random-effects meta-analysis with Hartung-Knapp-Sidik-Jonkman (HKSJ) adjusted 95% CIs is presented. The prediction interval is also shown [40-50]. AI: artificial intelligence; HKSJ: Hartung-Knapp-Sidik-Jonkman.

A total of 11 [40-50] RCTs involving 2220 participants (1091 in the experimental group and 1129 in the control group) were included in the meta-analysis. The pooled results indicated a small-to-moderate effect of AI-driven chatbots on depressive symptoms, compared with control conditions (SMD −0.46, 95% CI −1.02 to 0.10; *P*=.01; as shown in Figure 2). Negative SMD values indicate greater symptom reduction in the chatbot intervention groups.

To further quantify the real-world implications of heterogeneity, 95% PIs were calculated. The PI ranged from −1.50 to 0.58, indicating that the true effect in a future comparable setting could vary substantially and may include no effect. Heterogeneity among studies was substantial (*I*²=87%), suggesting considerable variability in intervention effects across trials.

Sensitivity analyses excluding Fitzpatrick et al [41] reduced heterogeneity from 87% to 60%, while the direction of the pooled effect remained unchanged. Detailed results are provided in Multimedia Appendix 3. To further explore potential sources of heterogeneity, subgroup analyses were conducted to examine differences across AI chatbot types.

### Overall Adherence of AI-Driven Chatbots in Depression Interventions

**Figure 3.**
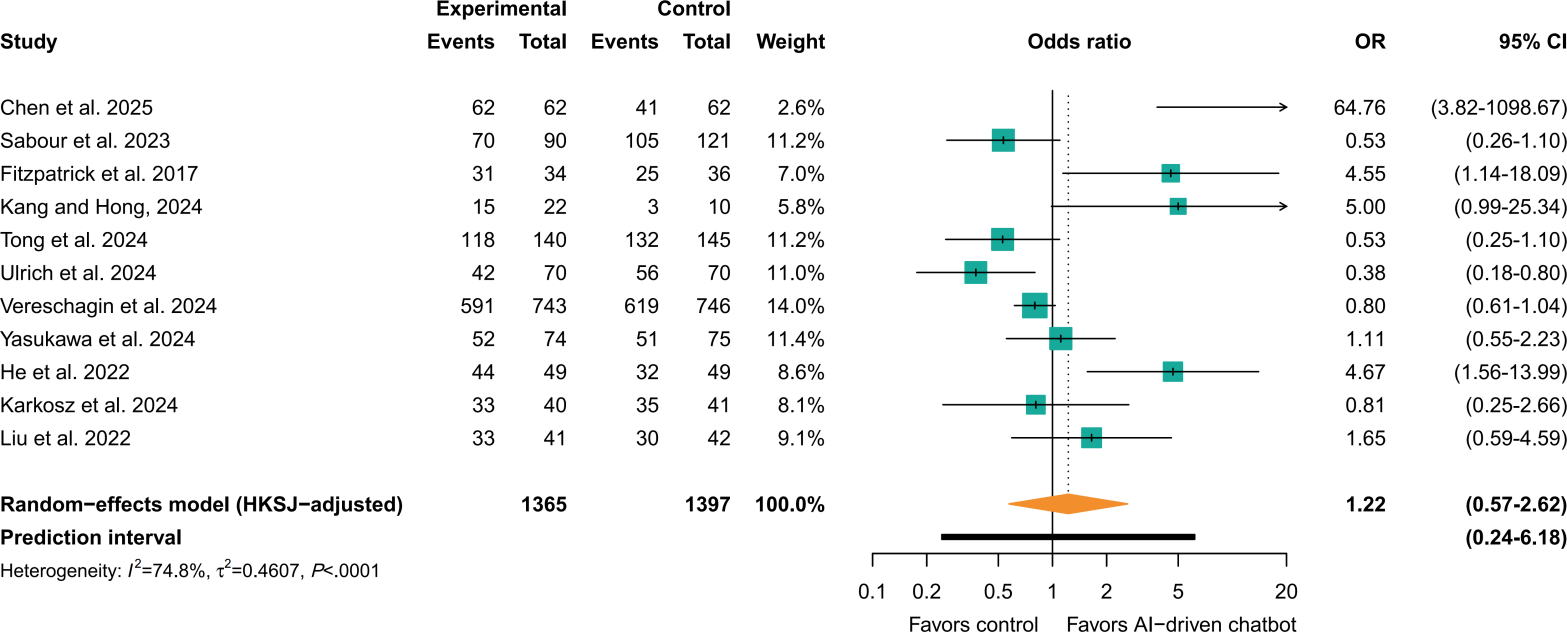
Forest plot of the overall effect of artificial intelligence (AI)–driven chatbots on user adherence in depression interventions. A random-effects meta-analysis with Hartung-Knapp-Sidik-Jonkman (HKSJ) adjusted 95% CIs is presented. The prediction interval is also shown [40-50]. AI: artificial intelligence; HKSJ: Hartung-Knapp-Sidik-Jonkman; OR: odds ratio.

Across all 11 [40-50] included studies, the pooled analysis showed no significant difference in adherence between intervention and control groups, as shown in Figure 3 (OR 1.22, 95% CI 0.57‐2.62; *P*=.57). To further interpret heterogeneity in real-world settings, the review calculated the 95% PI for the overall adherence outcome. The 95% PI ranged from 0.24 to 6.18, indicating substantial between-study variability. This suggests that the true adherence effect in a comparable future setting could range from lower to substantially higher engagement than in control conditions. Heterogeneity was considerable (*I*²=74.8%).

### Risk of Bias and Certainty of Evidence Assessment

**Figure 4.**
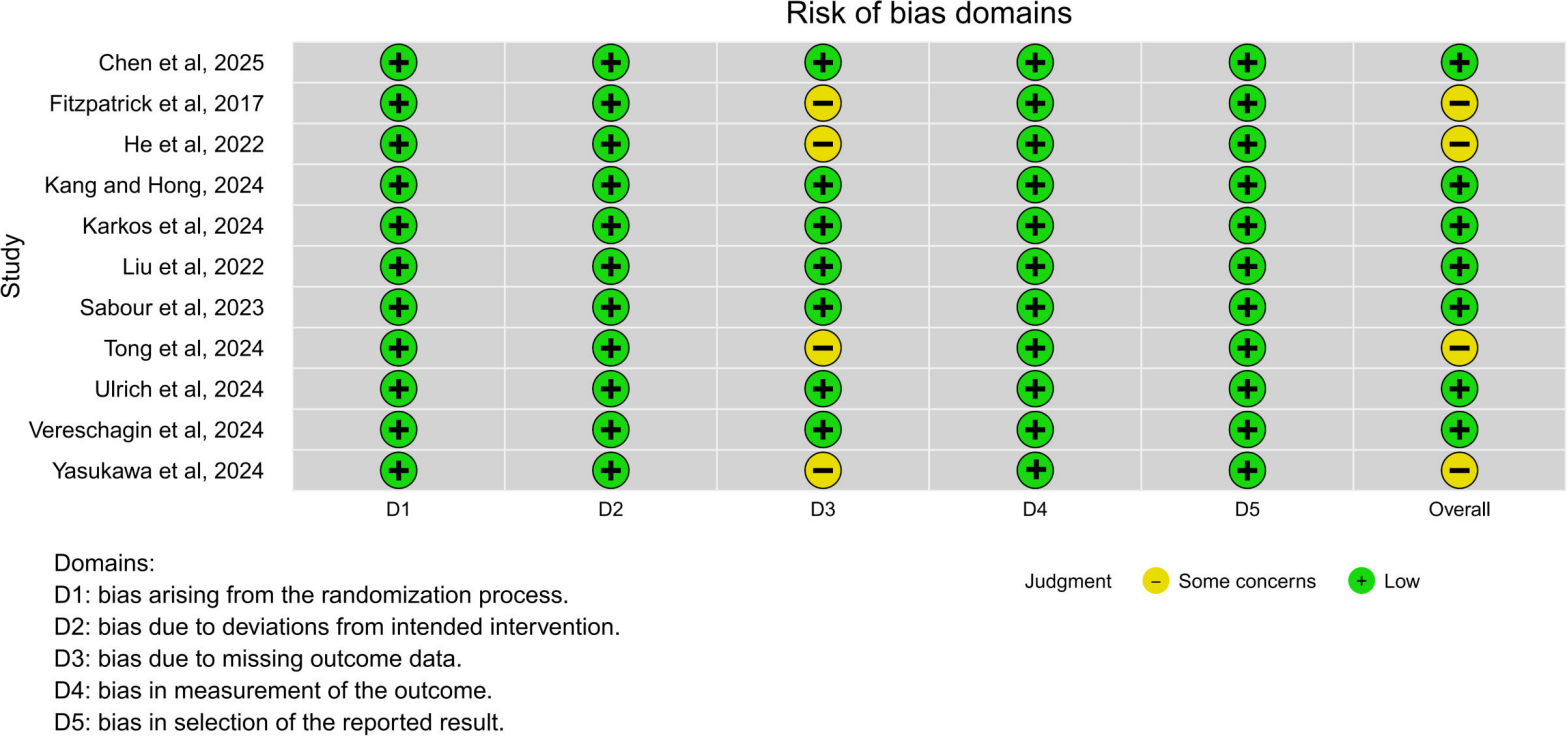
Risk of bias assessment of included studies using the Cochrane Risk of Bias (RoB) 2 tool [40-50].

All studies meeting the inclusion criteria reported depressive symptom outcomes at the end of the intervention, assessed using the PHQ-9. Overall, 7 studies [40,43-46,48,49] were judged to have a low risk of bias, while 4 studies [41,42,47,50] were assessed as having some concerns. The most common problem was missing outcome data (Domain 3), with insufficient reporting of outcomes in some studies. In contrast, all studies were judged to be of low risk across domains related to randomization, deviations from intended interventions, outcome measurement, and selective reporting. No study was considered to be at high risk of bias in any domain. Detailed domain-level assessments are presented in Figure 4 and Multimedia Appendix 4.

Certainty of evidence was assessed using the Grading of Recommendations Assessment, Development, and Evaluation (GRADE) approach. As all included studies were RCTs, the evidence started at high certainty. For clinical effectiveness, certainty was downgraded because of very serious inconsistency, serious imprecision, and suspected publication bias, resulting in very low-certainty evidence. For user adherence, certainty was downgraded because of serious inconsistency and serious imprecision, resulting in low-certainty evidence. A summary of the GRADE assessments for the main outcomes is presented in Table 2, and a more detailed GRADE evidence profile is provided in Multimedia Appendix 5.

**Table 2.** Grading of Recommendations Assessment, Development, and Evaluation (GRADE) summary of findings for the main outcomes of AI-driven chatbot interventions for depression.

Outcome	Number of studies (participants)	Effect estimate	Certainty of evidence	Reasons for downgrading
Clinical effectiveness (depressive symptom reduction)	11 RCTs^a^ (n=2220)	SMD^b^ –0.46 (95% CI –1.02 to 0.10)	Very low	Very serious inconsistency, serious imprecision, and suspected publication bias
User adherence	11 RCTs (n=2762)	OR^c^ 1.22 (95% CI 0.57-2.62)	Low	Serious inconsistency and serious imprecision

a RCT: randomized controlled trial

b SMD: standardized mean difference

c OR: odds ratio

### User Adherence Across Different AI-Driven Chatbot Types

Before presenting the subgroup analyses, this review clarifies the classification of AI-driven chatbots. Although LLMs are technically a subset of NLP, in this review we distinguish LLM-based chatbots as systems built on large pretrained generative models (eg, GPT, Gemini, and Claude) that directly generate responses in an open-ended way. In contrast, NLP-/ML-based chatbots are systems that use more traditional NLP or ML methods, such as intent classifiers, decision trees, or response selection within constrained conversational flows. Rule-based chatbots are systems that rely on fixed scripts or expert-crafted rules. This operational categorization aligns with how the primary studies describe their systems and is consistent with recent surveys that distinguish LLM paradigms from traditional NLP approaches [51,52].

**Figure 5.**
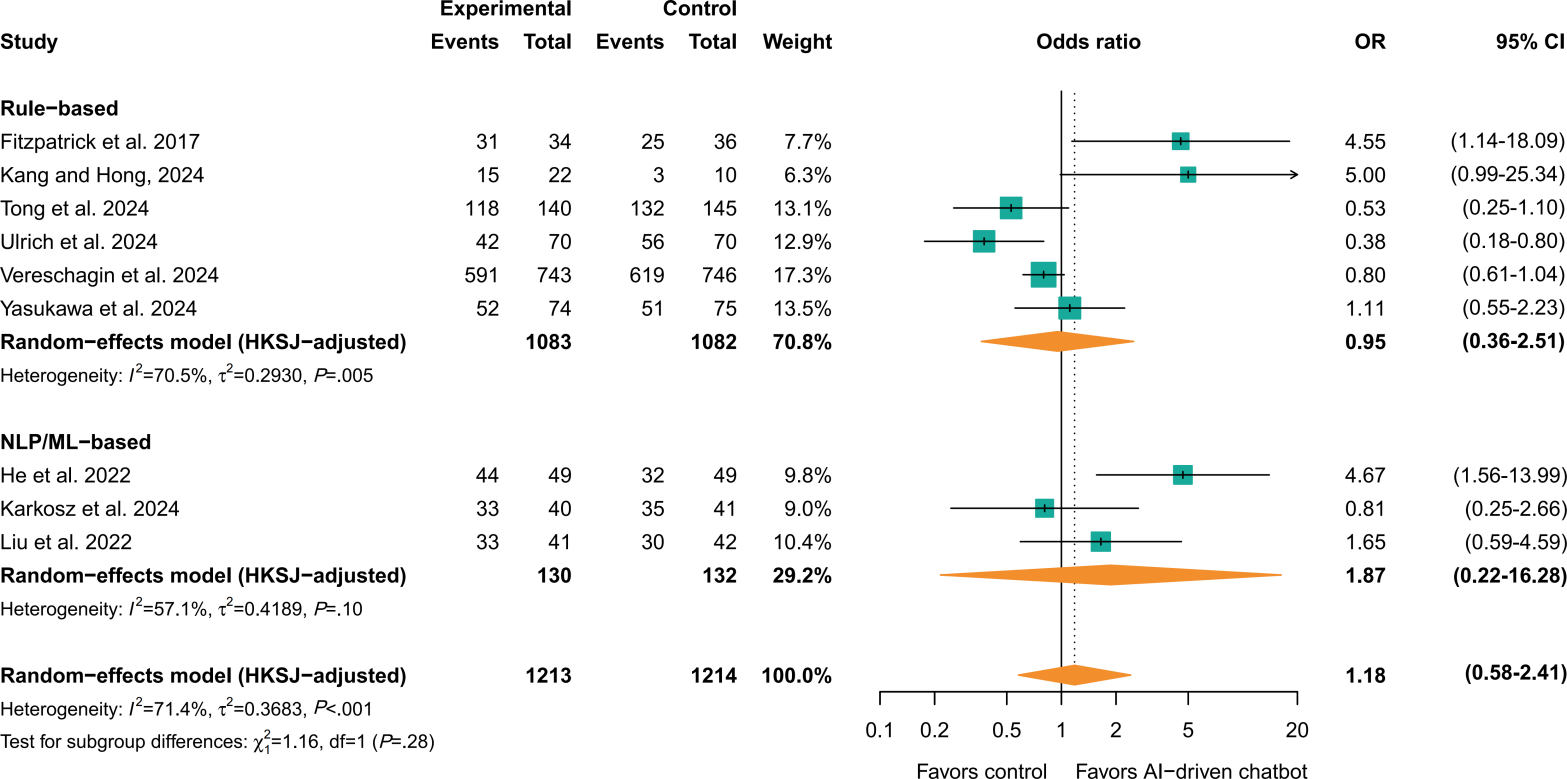
Subgroup analysis of user adherence to artificial intelligence (AI)–driven chatbot interventions for depression by AI type. Random-effects meta-analysis with Hartung-Knapp-Sidik-Jonkman (HKSJ) adjusted 95% CIs is presented for each subgroup. The large language model (LLM)–based subgroup was excluded because one included study reported complete adherence in the intervention group, resulting in an extreme and clinically uninterpretable pooled odds ratio estimate [41-45,47-49]. AI: artificial intelligence; HKSJ: Hartung-Knapp-Sidik-Jonkman; ML: machine learning; NLP: natural language processing; OR: odds ratio.

**Figure 6.**
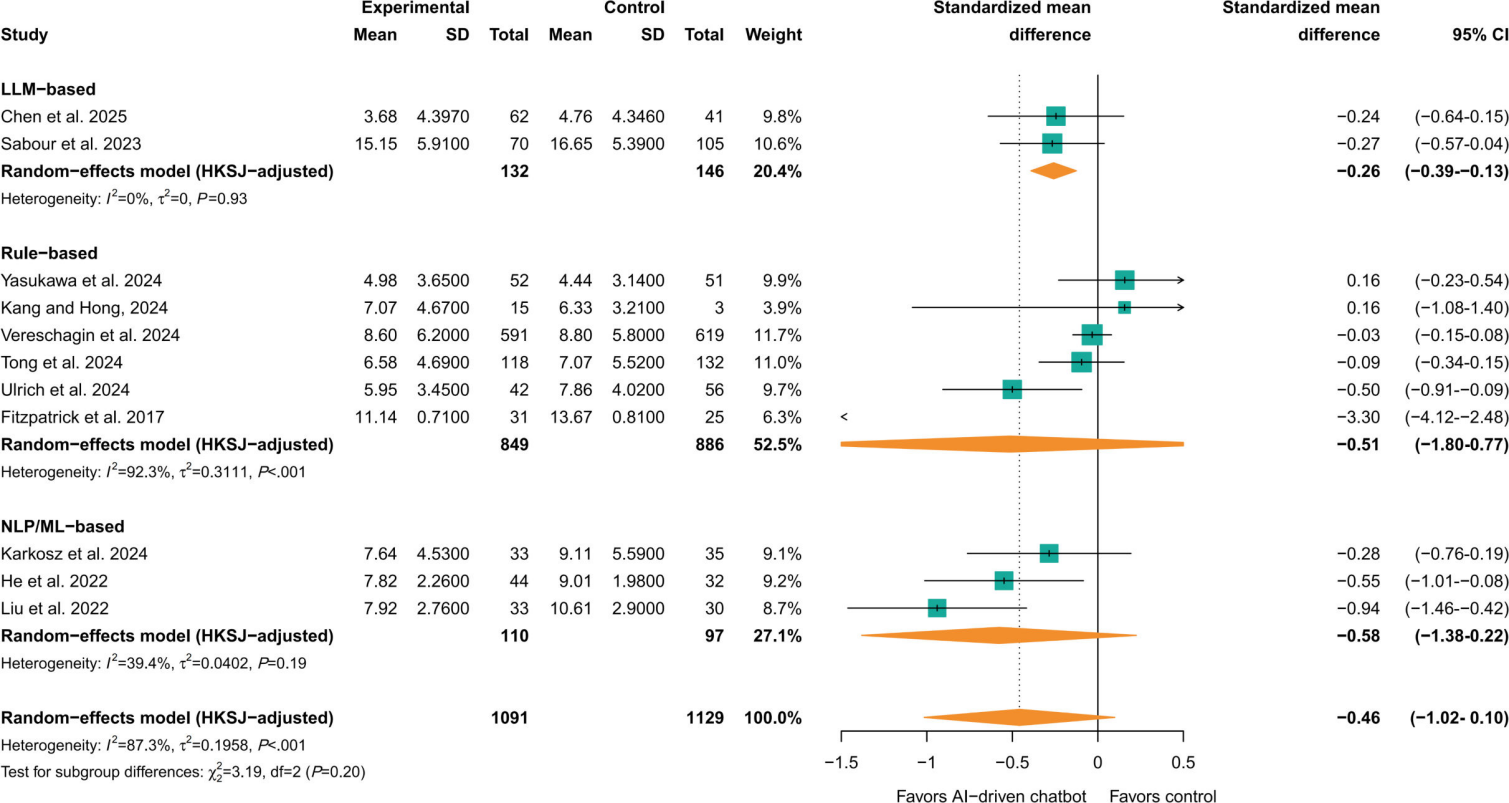
Subgroup analysis of the clinical effectiveness of artificial intelligence (AI)–driven chatbots in depression interventions by AI type. Random-effects meta-analysis with Hartung-Knapp-Sidik-Jonkman (HKSJ) adjusted 95% CIs is presented for each subgroup [40-50]. AI: artificial intelligence; HKSJ: Hartung-Knapp-Sidik-Jonkman; LLM: large language model; ML: machine learning; NLP: natural language processing.

To further explore heterogeneity in adherence, subgroup analyses were conducted by AI type (Figure 5; Figure 6). The pooled results indicated no significant overall difference in adherence between experimental and control groups (OR 1.22, 95% CI 0.57‐2.62; *P*=.57). To further interpret heterogeneity in real-world settings, we calculated the 95% PI for the overall adherence outcome. The 95% PI ranged from 0.24 to 6.18, indicating substantial between-study variability. This suggests that the true adherence effect in a comparable future setting could range from lower to substantially higher engagement than in control conditions.

At the subgroup level, no statistically significant effects were observed. Rule-based chatbots showed a nonsignificant pooled effect (OR 0.95, 95% CI 0.36‐2.51), while NLP/ML-based chatbots also did not reach statistical significance (OR 1.87, 95% CI 0.22‐16.28). The test for subgroup differences was not statistically significant (*P*=.47). The LLM-based subgroup was excluded from the adherence subgroup figure because one included study reported complete adherence in the intervention group, resulting in an extreme and clinically uninterpretable pooled OR estimate.

These findings suggest that chatbot type alone does not consistently predict user adherence across studies. Given the substantial heterogeneity, the results should be interpreted with caution. The findings further indicate that factors beyond chatbot type, such as specific interaction features, may play a more important role in sustaining user engagement. In the following section, we therefore turn to an analysis of 6 key interaction features, including interaction frequency, emotional responsiveness, self-disclosure encouragement, dialogue depth, feedback strategy, and user agency to clarify their contribution to adherence.

### Interaction Features in Relation to Clinical Effectiveness and User Adherence

This section examines 6 interaction features of AI-driven chatbots: interaction frequency, emotional responsiveness, self-disclosure encouragement, dialogue depth, feedback strategy, and user agency level. Studies were classified into high (≥3.75) and low (≤3.5) groups based on expert ratings. The detailed scoring table is provided in Multimedia Appendix 2. These ratings were derived from structured coding of published study descriptions and were used as operational proxies of interaction characteristics, rather than as direct measurements of chatbot behavior. The analyses in this section were therefore conducted in an exploratory, hypothesis-generating manner. Unlike the chatbot-type subgroup analysis presented earlier, the analyses in this section represent stratified meta-analyses based on study-level coding of interaction features. For each feature, studies were categorized as high or low according to predefined criteria, and pooled estimates were calculated separately within these strata. These analyses examine whether variation in interaction design characteristics is associated with differences in clinical effectiveness and user adherence, rather than comparing participant-level subgroups within individual trials. Section “Interaction Features and Clinical Effectiveness” reports the relationship between interaction features and clinical effectiveness, while section “Interaction Features and User Adherence” analyzes their association with user adherence.

### Interaction Features and Clinical Effectiveness

**Figure 7.**
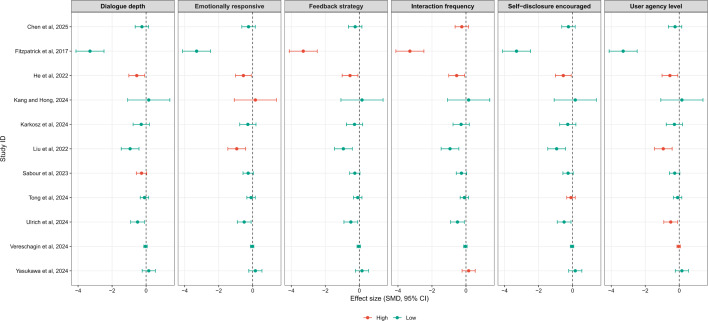
Stratified meta-analytic estimates of clinical effectiveness according to interaction feature level (high vs low study-level coding) [40-50]. SMD: standardized mean difference.

As shown in Figure 7 and summarized in Table 3, differences were observed between high and low-scoring subgroups across several interaction features. In general, high-scoring subgroups tended to show more consistent patterns of treatment effects, whereas low-scoring subgroups showed more heterogeneous and less stable estimates. These findings should be interpreted as exploratory contrasts rather than confirmatory evidence. Full model outputs and sensitivity analyses are provided in Multimedia Appendix 6 [40-50].

For dialogue depth, the high group was significantly associated with better outcomes (SMD=−0.35, 95% CI −0.61 to −0.10; *P*=.007; *I*²=0%), while the low group failed to reach significance and showed substantial heterogeneity (SMD=−0.54, 95% CI −1.20 to 0.11; *P*=.10; *I*²=97.0%). This pattern was largely influenced by the trial of Fitzpatrick et al [41], which used the Woebot platform and reported an exceptionally large effect size (SMD=−3.30, 95% CI −4.12 to −2.48). The authors noted frequent misunderstandings and repetitive dialogues as limitations, which may have shaped participants’ engagement and contributed substantially to the heterogeneity in this subgroup.

By contrast, He et al [42] was consistently classified into high groups across all 6 interaction features, with particularly high scores in emotional responsiveness (4.75). He et al [42] found that enhanced emotional awareness significantly predicted superior therapeutic outcomes (F_2, 145_=3.636; *P*=.03), a finding corroborated by the meta-analysis (SMD=−0.55, 95% CI −1.01 to −0.08; *P*=.02; *I*²=80%). This provides indicative evidence that empathetic and adaptive chatbot responses may enhance clinical effectiveness. In addition, Liu et al [45] was classified into the low group for interaction frequency (score=3). The intervention demonstrated a significant negative effect (SMD −0.94, 95% CI −1.46 to −0.42), suggesting that insufficient interaction intensity may limit sustained therapeutic gains.

Taken together, these exploratory findings suggest that dialogue depth, emotional responsiveness, and interaction frequency may be associated with variation in clinical effectiveness across studies. However, given the limited number of included trials and the substantial heterogeneity observed in several subgroups, these patterns should be interpreted with caution and regarded as hypothesis-generating rather than definitive evidence.

**Table 3.** Subgroup analyses of 6 interaction features and clinical effectiveness.

Interaction feature and subgroup	Pooled effect, SMD^a^ (95% CI)	Z^b^	*P* value	*I*^2^ (%)
Dialogue depth				
Low	–0.49 (–0.85 to –0.12)	2.63	.009	87
High	–0.35 (–0.61 to –0.10)	2.71	.007	0
Emotionally responsive				
Low	–0.42 (–0.76 to –0.09)	2.48	.01	90
High	–0.63 (–1.07 to –0.19)	2.81	.01	33
Feedback strategy				
Low	–0.21 (–0.38 to –0.04)	2.36	.02	59
High	–1.90 (–4.60 to –0.79)	1.98	.05	97
Interaction frequency				
Low	–0.26 (–0.48 to –0.05)	2.46	.01	65
High	–0.92 (–1.95 to 0.11)	1.74	.08	93
Self-disclosure encouraged				
Low	–0.52 (–0.90 to –0.14)	2.67	.008	90
High	–0.28 (–0.71 to 0.16)	1.25	.21	65
User agency level				
Low	–0.49 (–1.00 to 0.01)	1.92	.05	90
High	–0.46 (–0.90 to ‐0.03)	2.08	.04	84

a SMD: standardized mean difference.

b Z denotes the Wald test statistic used for pooled odds ratios.

### Interaction Features and User Adherence

**Figure 8.**
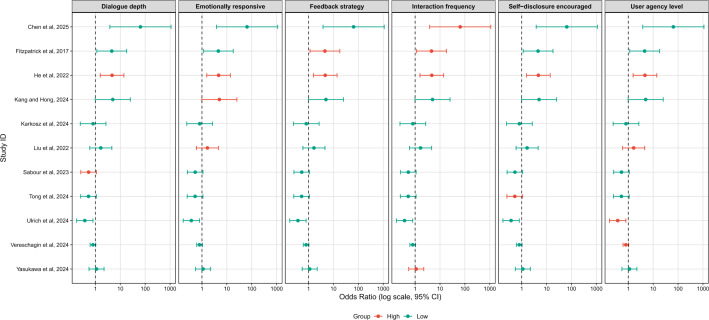
Stratified meta-analytic estimates of user adherence according to interaction feature level (high vs low study-level coding) [40-50].

Subgroup analyses were conducted to examine the association between 6 interaction features and user adherence, using the completion-based adherence definition described in the Methods (as shown in Figure 8 and Table 4). Overall, differences were observed between high- and low-scoring subgroups across several interaction features. In contrast to the clinical effectiveness outcomes reported in Figure 7, effect estimates for user adherence showed greater variability in magnitude and precision across studies.

For emotional responsiveness, the high-scoring subgroup showed a statistically significant association with adherence (OR 3.03, 95% CI 1.45‐6.36; *P*=.003; *I*²=14%), whereas the low-scoring subgroup did not reach statistical significance (OR 0.87, 95% CI 0.53‐1.44; *P*=.59; *I*²=70%). He et al [42], who were classified in the high group (score of 4.75), reported a statistically significant association between emotional awareness and adherence. He et al [42] illustrates how emotionally responsive chatbot interactions may be associated with adherence outcomes in specific contexts, rather than providing confirmatory evidence of a causal relationship.

In the domain of feedback strategy, high-scoring studies yielded robust and consistent associations (OR 4.62, 95% CI 1.96‐10.91; *P*<.001; *I*²=0%). Fitzpatrick et al [41] and He et al [42] both applied structured feedback mechanisms, and their findings largely drove the statistical significance of this subgroup. While these findings highlight a consistent pattern within the available data, they should be interpreted cautiously given the small number of contributing studies.

With regard to interaction frequency, the high group demonstrated a significant advantage (OR 4.18, 95% CI 1.10‐15.87; *P*=.04; *I*²=78%), whereas the low group did not (OR 0.75, 95% CI 0.49‐1.13; *P*=.17; *I*²=53%). Chen et al [40], and Yasukawa et al [50], which were included in the high-frequency subgroup, both reported patterns consistent with sustained adherence under more frequent interactions. Rather than implying a causal relationship, this contrast illustrates variability in adherence outcomes across different interaction intensity profiles.

**Table 4.** Subgroup analyses of 6 interaction features and user adherence.

Interaction feature and subgroup	Pooled effect, OR^a^ (95% CI)	Z^b^	*P* value	*I*^2^ (%)
Dialogue depth				
Low	1.17 (0.68-2.01)	0.56	.57	72
High	1.52 (0.18-12.80)	0.38	.70	91
Emotionally responsive				
Low	0.87 (0.53-1.44)	0.55	.59	70
High	3.03 (1.45-6.36)	2.93	.003	14
Feedback strategy				
Low	0.90 (0.57-1.44)	0.43	.67	67
High	4.62 (1.96-10.91)	3.50	<.001	0
Interaction frequency				
Low	0.75 (0.49-1.13)	1.38	.17	53
High	4.18 (1.10-15.87)	2.10	.04	78
Self-disclosure encouraged				
Low	1.17 (0.68-2.01)	0.56	.57	72
High	1.51 (0.18-12.83)	0.38	.70	91
User agency level				
Low	1.48 (0.65-3.35)	0.94	.35	76
High	1.11 (0.49-2.53)	0.25	.80	81

a OR: odds ratio.

b Z denotes the Wald test statistic used for pooled odds ratios.

Taken together, these exploratory results suggest that emotional responsiveness, feedback strategy, and interaction frequency may be associated with variation in adherence outcomes across studies. In contrast, self-disclosure encouragement, dialogue depth, and user agency level showed weaker or nonsignificant associations. Given the heterogeneity of adherence estimates and the limited number of included trials, these patterns should be interpreted as hypothesis-generating and contingent on contextual and design-specific factors.

### Small-Study Effects

Funnel plots were generated to assess potential small-study effects for the primary outcomes (Multimedia Appendix 7). For clinical effectiveness, visual inspection suggested possible funnel plot asymmetry. Egger regression test (*P*=.05) and the Begg-Mazumdar rank correlation test (*P*=.04) both indicated statistically significant asymmetry. The trim-and-fill procedure imputed 3 potentially missing studies, and the adjusted pooled estimate was attenuated (SMD=−0.13, 95% CI −0.47 to 0.21) and no longer statistically significant. However, substantial heterogeneity was present (*I*²=87%), and funnel plot asymmetry in this context may reflect genuine between-study variability rather than publication bias alone. For user adherence, statistical testing did not indicate significant funnel plot asymmetry (Begg test *P*=.07). Although the trim-and-fill method imputed 3 studies, the adjusted pooled estimate remained nonsignificant (OR 0.84, 95% CI 0.49‐1.45), suggesting that small-study effects did not materially alter the overall conclusion for adherence outcomes.

## Discussion

### Summary of Key Findings

This systematic review evaluated the clinical effectiveness of AI-driven chatbots for depression and examined how interaction features relate to treatment outcomes and user adherence. AI-driven chatbots tended to reduce depressive symptoms, but after a more conservative analysis, this effect lost statistical significance, and studies remained heterogeneous.

No stable or statistically significant differences in user adherence were observed across chatbot types. This suggests that chatbot type alone may not explain variation in engagement patterns across studies. In addition, user adherence and clinical effectiveness did not show a stable one-to-one relationship.

By contrast, the exploratory analyses of interaction features revealed more informative patterns. Emotional responsiveness, feedback strategy, and interaction frequency showed more consistent associations with user adherence, whereas their relationships with clinical effectiveness were more mixed and heterogeneous. Dialogue depth, self-disclosure encouragement, and user agency showed weaker or more context-dependent associations. Overall, these findings suggest that interaction design may offer more explanatory value than chatbot type alone in understanding variation across studies.

### Overall Interpretation

The findings of this review are broadly consistent with previous research on DMHIs. Previous studies suggest that technology-assisted interventions may reduce depressive symptoms to some extent, but their effect sizes are often small to moderate and vary across studies [53-55]. A similar pattern was observed in the present review. Although the pooled effect on depressive symptoms remained in a favorable direction, it was no longer statistically significant after applying the more conservative HKSJ method, and substantial heterogeneity remained. The wide PI further suggests that the true effect in a comparable future setting could vary considerably, potentially including no meaningful benefit. Taken together, these findings suggest that the overall clinical effectiveness of AI-driven chatbot interventions remains uncertain and may be influenced by differences in intervention design, implementation context, and study populations [56,57]. This cautious interpretation is also consistent with the GRADE assessment, which rated the certainty of evidence for clinical effectiveness as very low.

Another important finding of this review is that user adherence and clinical effectiveness did not show a stable or directly corresponding relationship. In this review, some studies reported relatively high levels of sustained engagement at the descriptive level [46,48,50], but this pattern did not consistently correspond to greater overall symptom improvement. This finding is consistent with previous research showing that user engagement is an important condition for the success of digital interventions, but it does not reliably predict clinical benefit [58,59]. In the present review, the overall adherence analysis also showed no significant difference between intervention and control groups. Heterogeneity remained high, and the PI was wide. This suggests that adherence outcomes may also vary substantially across studies and implementation settings. In this sense, adherence may be understood as an important condition for intervention success but not a sufficient one [55,59]. At the same time, the findings of this review suggest that chatbot type alone does not provide a stable explanation for variation in effectiveness or adherence across studies.

Building on this distinction, the present review further examined how different interaction features may relate to these divergent patterns. The exploratory analyses suggest that different interaction design elements may relate differently to user engagement and symptom change. In particular, the associations between interaction features and user adherence appeared to be clearer, whereas their relationships with clinical effectiveness were more mixed and heterogeneous. Taken together, these findings suggest that variation across studies may be better understood by focusing on interaction design features rather than chatbot type alone. In other words, differences across studies may be more closely related to how interactions are designed and implemented than to the underlying technical category itself [55,56,59]. At the same time, given the low certainty of evidence for the main outcomes, these interpretations should remain cautious. A more fine-grained understanding of interaction design may help explain variability across studies and inform future system development.

### Potential Design Implications of Interaction Features

The exploratory analyses suggested that interaction features may provide a more informative lens than chatbot type alone for understanding variation across studies. To clarify these findings, the potential design implications are discussed separately for clinical effectiveness and user adherence. Given the heterogeneity of the evidence and the low certainty of the main outcomes, these implications should be understood as cautious and exploratory rather than prescriptive design recommendations.

### Interaction Features and Clinical Effectiveness

The exploratory analyses suggested that the relationships between interaction features and clinical effectiveness were mixed and heterogeneous. Across the 6 interaction features, no single feature showed a uniformly stable association with symptom improvement. Instead, different features appeared to relate to treatment outcomes in different ways, and their potential value seemed to depend on intervention context, therapeutic structure, and user characteristics. Taken together, these findings suggest that interaction design may contribute to clinical effectiveness, but the current evidence does not support simple or universal design conclusions.

Among the 6 features, dialogue depth showed one of the clearest patterns in relation to treatment outcomes. Deeper dialogue was associated with more consistent symptom improvement, whereas lower dialogue depth was linked to more variable treatment effects. These findings suggest that dialogue depth may support therapeutic benefit when it is appropriately structured. Existing research provides mixed evidence regarding the value of open-ended dialogue in DMHIs [51]. Many AI-driven chatbots rely on structured and guided conversational flows to maintain clarity and therapeutic focus [12]. While more open dialogue may increase perceived empathy and human-likeness [52], it may also increase cognitive load or lead to topic drift if not carefully designed [60]. Evidence from cross-cultural studies suggests that reflective and emotionally expressive dialogue can be beneficial, but its impact depends on contextual relevance and timing [61]. From a design perspective, dialogue depth may be better understood as an adaptive feature rather than a fixed attribute [62-64]. Integrating deeper dialogue within structured therapeutic components, such as journaling or behavioral activation tasks, may help maintain alignment with therapeutic objectives [65]. Overall, these observations highlight an important tension between expressive interaction and cognitive manageability. Dialogue depth may contribute to therapeutic alliance and perceived empathy under certain conditions [66], but its effectiveness likely depends on user characteristics, emotional state, and intervention structure.

Other interaction features showed less consistent relationships with symptom change. Emotional responsiveness was more consistently associated with user adherence and, to a lesser extent, with clinical outcomes. Interventions that incorporated more consistent and contextually appropriate emotional feedback tended to show more stable effects, but increasing emotional expressiveness without moderation is unlikely to improve outcomes directly. Research suggests that user engagement is influenced not only by emotional tone but also by the relevance and structure of therapeutic content [67]. Experimental work further indicates that improvements in emotional response mechanisms may enhance user trust and cognitive restructuring processes [68]. Emotional responsiveness may therefore be better understood as a process of calibration rather than intensity [69,70].

Feedback strategies also appeared to have a less stable relationship with clinical effectiveness than with adherence. Interventions that incorporated structured and personalized feedback tended to show more stable engagement patterns, but their influence on symptom improvement likely depends on how feedback is implemented and integrated within the intervention. Within internet-delivered cognitive behavioral therapy, individualized feedback has been associated with lower dropout even when symptom change is comparable [71]. This suggests that feedback may support treatment delivery, but its direct clinical impact is likely to vary across contexts.

Interaction frequency similarly showed different patterns for symptom change and sustained engagement. Lower-frequency interventions were associated with more consistent symptom improvement, whereas higher-frequency contact did not necessarily correspond to better short-term outcomes. Some studies have found that increased conversational exchange is associated with symptom improvement [72], while others report that gains may stabilize or diminish over longer periods of exposure [73]. This suggests that the effects of interaction frequency may not be linear. From a design perspective, interaction frequency should therefore be considered alongside timing, tone, and user context, and adaptive scheduling may be preferable to fixed high-frequency contact.

By comparison, self-disclosure encouragement and user agency showed weaker and more context-dependent relationships with clinical effectiveness. Encouraging self-disclosure was not consistently associated with improvements in symptoms. This contrasts with prior evidence indicating that self-disclosure is a key mechanism for building therapeutic alliance and enhancing engagement [74-76]. In face-to-face care, disclosure helps reduce stigma and promotes help-seeking, and digital interventions have attempted to replicate these processes through structured prompts for emotional expression and narrative sharing [77,78]. Taken together, these findings suggest that self-disclosure may not function in the same way across digital and in-person settings. Similarly, user agency was not consistently associated with clinical outcomes. Previous research supports the importance of perceived control in digital mental health systems [41,79], but agency may shape how users experience and engage with the intervention rather than directly improve outcomes.

Taken together, the exploratory findings suggest that interaction features may contribute to clinical effectiveness, but their relationships with symptom improvement are mixed and strongly shaped by context. Dialogue depth appeared to show the clearest potential relevance to therapeutic benefit, whereas emotional responsiveness, feedback strategy, and interaction frequency showed less stable associations with clinical outcomes. Self-disclosure encouragement and user agency showed weaker and more context-dependent patterns. These observations suggest that potential design implications for clinical effectiveness should be interpreted cautiously. At present, the evidence is better suited to generating conceptual implications than to supporting fixed design recommendations.

### Interaction Features and User Adherence

The exploratory analyses suggested that interaction features showed clearer and more consistent patterns for user adherence than for clinical effectiveness. Across the 6 interaction features, emotional responsiveness, feedback strategy, and interaction frequency appeared to be more consistently associated with sustained engagement, whereas dialogue depth, self-disclosure encouragement, and user agency showed weaker or more context-dependent relationships. Taken together, these findings suggest that interaction design may be particularly important for understanding continued participation in chatbot-based interventions.

Emotional responsiveness showed one of the clearest relationships with user adherence. Interventions that incorporated more consistent and contextually appropriate emotional feedback tended to show more stable effects. However, this does not suggest that increasing emotional expressiveness without moderation will necessarily improve outcomes. Rather, emotional responsiveness may support engagement when it is calibrated appropriately, whereas excessive or poorly timed amplification may increase emotional burden or cognitive load and thereby undermine sustained engagement [80-82]. Qualitative studies have shown that users value personalized emotional support delivered at an appropriate pace [83]. Concerns about fully automated systems often extend beyond privacy and safety to include whether the system responds in a socially and emotionally appropriate manner [84]. Personalization therefore remains important, and adjusting tone, timing, and response frequency in relation to recent mood patterns may enhance usability and satisfaction [85].

Feedback strategy also appeared to play an important role in supporting user adherence. Interventions that incorporated structured and personalized feedback tended to show more stable engagement patterns. Timely and context-aware prompts can increase short-term engagement [86], whereas generic reminders may be less effective for sustaining engagement in real-world settings [87]. Broader research on guided digital interventions indicates that formats incorporating responsive elements or human support tend to achieve better retention than unguided approaches [88-90]. Methodological reviews further identify adherence and attrition as central determinants of overall effectiveness in DMHIs [91,92]. Feedback may therefore serve as a reinforcement cue that helps stabilize engagement over time, although excessive or poorly timed prompts may contribute to notification fatigue [93].

Interaction frequency similarly showed a clearer relationship with sustained engagement than with symptom change. Higher-frequency contact appeared more closely linked to continued participation. Interaction frequency may operate as both a structural and behavioral cue. A predictable rhythm of contact can reduce decision burden and support habit formation by transforming prompts into routine action cues [94-96]. From a design perspective, interaction frequency should be considered alongside timing, tone, and user context. Adaptive scheduling based on user behavior or mood patterns may be preferable to fixed high-frequency contact, and allowing users to adjust contact frequency may further support autonomy and reduce fatigue.

By comparison, dialogue depth showed a less stable relationship with sustained engagement. Although deeper dialogue was associated with more consistent symptom improvement, its relationship with continued use was less clear, and lower dialogue depth was linked to inconsistent adherence patterns. Existing research provides mixed evidence regarding the value of open-ended dialogue in DMHIs [51]. Many AI-driven chatbots rely on structured and guided conversational flows to maintain clarity and therapeutic focus [12]. While more open dialogue may increase perceived empathy and human-likeness [52], it may also increase cognitive load or lead to topic drift if not carefully designed [60]. Evidence from cross-cultural studies suggests that reflective and emotionally expressive dialogue can be beneficial, but its impact depends on contextual relevance and timing [61]. Research on digital behavior change interventions also suggests that early interactions should minimize cognitive demands to support initial engagement [97], and providing users with options to regulate conversational depth may reduce interaction fatigue [98].

Encouraging self-disclosure and increasing user agency also showed weaker and more context-dependent relationships with adherence. Although prior evidence indicates that self-disclosure can support therapeutic alliance and engagement [74-76], the present findings suggest that its effects in digital interventions may depend more strongly on timing, pacing, and context. Digital interventions have attempted to introduce structured prompts for emotional expression and narrative sharing [77,78], but willingness to disclose sensitive information may vary across regions and populations [99,100]. Privacy and ethical concerns remain important barriers, as fear of data misuse or personal information leakage can directly undermine trust and weaken adherence [101-103]. Similarly, user agency was not consistently associated with sustained engagement, although providing an appropriate degree of choice and control may still contribute to perceived engagement and satisfaction. Previous research supports the importance of perceived control in digital mental health systems [79]. At the same time, excessive freedom may increase interactional burden, whereas overly constrained interaction may reduce perceived control and engagement [104]. A balanced approach may therefore be more acceptable across different users and contexts [105].

Taken together, the exploratory findings suggest that interaction features may offer greater explanatory value for user adherence than for clinical effectiveness. In particular, emotional responsiveness, feedback strategy, and interaction frequency appeared to be more consistently related to sustained engagement, whereas dialogue depth, self-disclosure encouragement, and user agency seemed more dependent on timing, structure, and user readiness. However, given the heterogeneity of the evidence and the low certainty of the main outcomes, these patterns should be interpreted cautiously as conceptual implications rather than prescriptive design rules.

### Limitations

This systematic review has several limitations that should be considered when interpreting the findings. First, the number of included studies was relatively small (n=11), particularly for subgroup analyses by AI chatbot type. This may have limited statistical power and reduced the generalizability of the findings. Second, substantial heterogeneity was observed across studies. Variations in intervention duration, chatbot design, delivery format, and target populations may have contributed to the variability and uncertainty in effect estimates, despite the use of random-effects models. The wide PIs in the main analyses further suggest that effects may vary across comparable future settings.

Third, interaction features were extracted and scored based on descriptions reported in the included studies rather than direct inspection of chatbot behavior. Although a structured coding protocol was applied and ratings were conducted independently by 2 human-computer interaction experts, with adjudication by a third expert, some degree of subjectivity in feature interpretation was unavoidable. The feature-level findings should therefore be interpreted as exploratory. Finally, this meta-analysis relied exclusively on published studies, which may have introduced publication bias, as studies reporting nonsignificant or negative results are less likely to be published. In addition, statistical assessment indicated evidence of small-study effects for clinical effectiveness, and trim-and-fill adjustment attenuated the pooled estimate. However, given the substantial heterogeneity across studies, funnel plot asymmetry may partly reflect genuine between-study variability rather than publication bias alone. Taken together, these limitations indicate that the main findings should be interpreted cautiously.

### Future Directions

Future research should adopt more standardized reporting of interaction features, clinical outcomes, and adherence measures. Studies that directly test the causal impact of specific interaction strategies, ideally within comparable therapeutic frameworks, are needed to clarify how different design elements relate to symptom change and sustained engagement. In addition, adaptive and personalized interaction models warrant further investigation to better accommodate diverse user needs, intervention contexts, and patterns of use. More transparent reporting of chatbot interaction design and more standardized documentation of intervention characteristics would also improve comparability across studies and support stronger evidence synthesis in the future.

### Conclusion

The main contribution of this systematic review and meta-analysis is that it not only evaluated the clinical effectiveness of AI-driven chatbots for depression but also examined user adherence and interaction features within the same analytic framework. This allowed the review to move beyond the question of whether AI-driven chatbots may work and to explore possible reasons why findings vary across studies. The results showed a favorable trend for depressive symptom reduction, but the overall evidence remained uncertain. In addition, chatbot type alone did not provide a stable explanation for differences in user adherence. By contrast, interaction features, especially those related to sustained participation, appeared to offer a more informative perspective for understanding user engagement. Compared with previous reviews that mainly focused on overall effectiveness or differences between chatbot types, this study places greater emphasis on the role of interaction design in explaining variation in both outcomes and adherence. In this way, it offers a more fine-grained interpretive framework for the field. In practical terms, the findings suggest that the value of AI-driven chatbots for depression depends not only on the underlying technical architecture but also on how interactions are designed, structured, and supported over time. Future system development, clinical evaluation, and real-world implementation should therefore consider clinical outcomes and sustained engagement together, rather than relying only on short-term symptom change or chatbot type as the main basis for evaluation.

## Supplementary material

10.2196/88697 Multimedia Appendix 1 Search strategy.

10.2196/88697 Multimedia Appendix 2 Ratings of chatbot interaction features across included studies.

10.2196/88697 Multimedia Appendix 3 Leave-one-out sensitivity analysis of between-study heterogeneity (*I*²).

10.2196/88697 Multimedia Appendix 4 Risk of Bias (RoB) 2.

10.2196/88697 Multimedia Appendix 5 Grading of Recommendations Assessment, Development, and Evaluation (GRADE) summary.

10.2196/88697 Multimedia Appendix 6 Sensitivity analyses using the Hartung-Knapp-Sidik-Jonkman adjustment.

10.2196/88697 Multimedia Appendix 7 Funnel plot.

10.2196/88697 Checklist 1 PRISMA-S checklist.

10.2196/88697 Checklist 2 PRISMA checklist

## Abbreviations

AI: artificial intelligence
DMHI: digital mental health intervention
GRADE: Grading of Recommendations Assessment, Development, and Evaluation
HKSJ: Hartung-Knapp-Sidik-Jonkman
iCBT: internet-based cognitive behavioral therapy
LLM: large language model
ML: machine learning
NLP: natural language processing
OR: odds ratio
PHQ-9: Patient Health Questionnaire-9
PI: prediction interval
PRISMA: Preferred Reporting Items for Systematic Reviews and Meta-Analyses
PRISMA-S: Preferred Reporting Items for Systematic Reviews and Meta-Analyses Literature Search Extension
RCT: randomized controlled trial
RoB 2: Risk of Bias tool version 2
RQ: research question
SMD: standardized mean difference
WHO: World Health Organization
