# Supplementary material for: Therapeutic Interaction Features of AI Chatbots in Depression Interventions: Systematic Review and Meta-Analysis
Source: J Med Internet Res. 2026 Jun 30;28:e88697. doi: 10.2196/88697 (PMC13318397; doi:10.2196/88697)
Supplement: Multimedia Appendix 1 [file jmir-v28-e88697-s001.docx]

**Supplementary material 1**

**Search strategy**

Final search run date: 1 April 2026

Search coverage: database inception to 30 May 2025

1. **Web of science (808)**

TS=(

(depress* OR "major depressive disorder" OR dysthymi* OR "depressive symptom*" OR "mood disorder*" OR "affective disorder*")

AND

(chatbot* OR "conversational agent*" OR "dialogue system*" OR "virtual agent*" OR "virtual therapist*" OR Woebot OR Wysa OR Tess)

AND

(intervention* OR therap* OR treatment* OR counsel* OR psychotherap* OR "cognitive behavioral therapy" OR CBT OR "digital mental health" OR "digital therap*" OR "psychological intervention*")

)

TS=(

(

depress*

OR "major depressive disorder"

OR "MDD"

OR dysthymi*

OR "persistent depressive disorder"

OR "depressive disorder*"

OR "depressive symptom*"

OR "depressive episode*"

OR "recurrent depressive disorder"

OR "unipolar depression"

OR "mood disorder*"

OR "affective disorder*"

OR "subclinical depression"

OR "subthreshold depression"

)

AND

(

chatbot*

OR "chat bot*"

OR "conversational agent*"

OR "conversational AI"

OR "dialogue system*"

OR "dialog system*"

OR "virtual therapist*"

OR "virtual agent*"

OR "relational agent*"

OR "embodied agent*"

OR "mental health bot*"

OR Woebot

OR Wysa

OR Tess

OR Youper

OR Replika

OR Ellie

)

)

1. **Scopus (1040)**

TITLE-ABS-KEY(

(

depress*

OR "major depressive disorder"

OR MDD

OR dysthymi*

OR "depressive symptom*"

OR "mood disorder*"

OR "affective disorder*"

)

AND

(

chatbot*

OR "AI chatbot*"

OR "artificial intelligence chatbot*"

OR "conversational agent*"

OR "dialogue system*"

OR "virtual agent*"

OR "virtual therapist*"

OR "mental health chatbot*"

OR "digital conversational agent*"

OR Woebot

OR Wysa

OR Tess

)

AND

(

intervention*

OR therap*

OR treatment*

OR counsel*

OR psychotherap*

OR "cognitive behavioral therapy"

OR CBT

OR "digital mental health"

OR "digital therap*"

OR "psychological intervention*"

OR "clinical trial*"

OR "randomized controlled trial"

OR "randomised controlled trial"

OR RCT

)

)

TITLE-ABS-KEY(

(

depress*

OR "major depressive disorder"

OR "MDD"

OR dysthymi*

OR "persistent depressive disorder"

OR "depressive disorder*"

OR "depressive symptom*"

OR "depressive episode*"

OR "recurrent depressive disorder"

OR "unipolar depression"

OR "mood disorder*"

OR "affective disorder*"

OR "subclinical depression"

OR "subthreshold depression"

)

AND

(

chatbot*

OR "chat bot*"

OR "conversational agent*"

OR "conversational AI"

OR "dialogue system*"

OR "dialog system*"

OR "virtual therapist*"

OR "virtual agent*"

OR "relational agent*"

OR "embodied agent*"

OR "mental health bot*"

OR Woebot

OR Wysa

OR Tess

OR Youper

OR Replika

OR Ellie

)

)

1. **IEEE (249)**

(

depress*

OR "major depressive disorder"

OR MDD

OR dysthymi*

OR "depressive disorder"

OR "depressive symptoms"

OR "mood disorder"

)

AND

(

chatbot

OR chatbots

OR "AI chatbot"

OR "conversational agent"

OR "dialogue system"

OR "virtual agent"

OR "intelligent agent"

OR "dialogue agent"

OR Woebot

OR Wysa

OR Tess

)

AND

(

intervention

OR therapy

OR treatment

OR psychotherapy

OR counseling

OR counselling

OR "cognitive behavioral therapy"

OR CBT

)

AND

(

random*

OR trial

OR "controlled study"

OR RCT

)

("All Metadata":"depression"

OR "All Metadata":"depressed"

OR "All Metadata":"depressive"

OR "All Metadata":"major depressive disorder"

OR "All Metadata":"MDD"

OR "All Metadata":"dysthymia"

OR "All Metadata":"dysthymic"

OR "All Metadata":"persistent depressive disorder"

OR "All Metadata":"depressive disorder"

OR "All Metadata":"depressive disorders"

OR "All Metadata":"depressive symptoms"

OR "All Metadata":"depressive episode"

OR "All Metadata":"unipolar depression"

OR "All Metadata":"mood disorder"

OR "All Metadata":"mood disorders"

OR "All Metadata":"affective disorder"

OR "All Metadata":"affective disorders"

OR "All Metadata":"subclinical depression"

OR "All Metadata":"subthreshold depression")

AND

("All Metadata":"chatbot"

OR "All Metadata":"chatbots"

OR "All Metadata":"chat bot"

OR "All Metadata":"chat bots"

OR "All Metadata":"conversational agent"

OR "All Metadata":"conversational agents"

OR "All Metadata":"conversational AI"

OR "All Metadata":"dialogue system"

OR "All Metadata":"dialogue systems"

OR "All Metadata":"dialog system"

OR "All Metadata":"dialog systems"

OR "All Metadata":"virtual therapist"

OR "All Metadata":"virtual therapists"

OR "All Metadata":"virtual agent"

OR "All Metadata":"virtual agents"

OR "All Metadata":"relational agent"

OR "All Metadata":"relational agents"

OR "All Metadata":"embodied agent"

OR "All Metadata":"embodied agents"

OR "All Metadata":"mental health bot"

OR "All Metadata":"mental health bots"

OR "All Metadata":"Woebot"

OR "All Metadata":"Wysa"

OR "All Metadata":"Tess"

OR "All Metadata":"Youper"

OR "All Metadata":"Replika"

OR "All Metadata":"Ellie")

1. **PubMed (379)**

(

("Depressive Disorder"[Mesh]

OR "Depression"[Mesh]

OR depress*[tiab]

OR "major depressive disorder"[tiab]

OR MDD[tiab]

OR dysthymi*[tiab]

OR "depressive symptom*"[tiab]

OR "mood disorder*"[tiab]

OR "affective disorder*"[tiab]

)

AND

(

"Chatbots"[Mesh]

OR chatbot*[tiab]

OR "AI chatbot*"[tiab]

OR "artificial intelligence chatbot*"[tiab]

OR "conversational agent*"[tiab]

OR "dialogue system*"[tiab]

OR "virtual agent*"[tiab]

OR "virtual therapist*"[tiab]

OR "mental health chatbot*"[tiab]

OR "digital conversational agent*"[tiab]

OR Woebot[tiab]

OR Wysa[tiab]

OR Tess[tiab]

)

AND

(

"Psychotherapy"[Mesh]

OR "Cognitive Behavioral Therapy"[Mesh]

OR intervention*[tiab]

OR therap*[tiab]

OR treatment*[tiab]

OR counsel*[tiab]

OR psychotherap*[tiab]

OR "cognitive behavioral therapy"[tiab]

OR CBT[tiab]

OR "digital mental health"[tiab]

OR "digital therap*"[tiab]

OR "psychological intervention*"[tiab]

)

AND

(

"Randomized Controlled Trial"[Publication Type]

OR "Controlled Clinical Trial"[Publication Type]

OR randomized[tiab]

OR randomised[tiab]

OR RCT[tiab]

)

)

(

"Depression"[MeSH Terms]

OR "Depressive Disorder"[MeSH Terms]

OR "Depressive Disorder, Major"[MeSH Terms]

OR "Dysthymic Disorder"[MeSH Terms]

OR "Mood Disorders"[MeSH Terms]

OR depress*[tiab]

OR "major depressive disorder"[tiab]

OR "MDD"[tiab]

OR dysthymi*[tiab]

OR "persistent depressive disorder"[tiab]

OR "depressive disorder"[tiab]

OR "depressive disorders"[tiab]

OR "depressive symptom"[tiab]

OR "depressive symptoms"[tiab]

OR "depressive episode"[tiab]

OR "depressive episodes"[tiab]

OR "recurrent depressive disorder"[tiab]

OR "unipolar depression"[tiab]

OR "mood disorder"[tiab]

OR "mood disorders"[tiab]

OR "affective disorder"[tiab]

OR "affective disorders"[tiab]

OR "subclinical depression"[tiab]

OR "subthreshold depression"[tiab]

)

AND

(

chatbot[tiab]

OR chatbots[tiab]

OR "chat bot"[tiab]

OR "chat bots"[tiab]

OR "conversational agent"[tiab]

OR "conversational agents"[tiab]

OR "conversational AI"[tiab]

OR "dialogue system"[tiab]

OR "dialogue systems"[tiab]

OR "dialog system"[tiab]

OR "dialog systems"[tiab]

OR "virtual therapist"[tiab]

OR "virtual therapists"[tiab]

OR "virtual agent"[tiab]

OR "virtual agents"[tiab]

OR "relational agent"[tiab]

OR "relational agents"[tiab]

OR "embodied agent"[tiab]

OR "embodied agents"[tiab]

OR "mental health bot"[tiab]

OR "mental health bots"[tiab]

OR Woebot[tiab]

OR Wysa[tiab]

OR Tess[tiab]

OR Youper[tiab]

OR Replika[tiab]

OR Ellie[tiab]

)

1. **PsycINFO (202)**

(

exp Depressive Disorder/

OR exp Depression/

OR depress*.ti,ab.

OR "major depressive disorder".ti,ab.

OR MDD.ti,ab.

OR dysthymi*.ti,ab.

OR "depressive symptom*".ti,ab.

OR "mood disorder*".ti,ab.

OR "affective disorder*".ti,ab.

)

AND

(

exp Chatbots/

OR chatbot*.ti,ab.

OR "AI chatbot*".ti,ab.

OR "conversational agent*".ti,ab.

OR "dialogue system*".ti,ab.

OR "virtual agent*".ti,ab.

OR "virtual therapist*".ti,ab.

OR Woebot.ti,ab.

OR Wysa.ti,ab.

OR Tess.ti,ab.

)

AND

(

exp Psychotherapy/

OR exp Cognitive Behavior Therapy/

OR intervention*.ti,ab.

OR therap*.ti,ab.

OR treatment*.ti,ab.

OR counsel*.ti,ab.

OR psychotherap*.ti,ab.

OR "cognitive behavioral therapy".ti,ab.

OR CBT.ti,ab.

OR "digital mental health".ti,ab.

OR "digital therap*".ti,ab.

OR "psychological intervention*".ti,ab.

)

AND

(

random*.ti,ab.

OR randomized.ti,ab.

OR randomised.ti,ab.

OR RCT.ti,ab.

OR "controlled trial".ti,ab.

)

Block A — Depression (Lines 1–18)

1. exp depression/

2. exp depressive disorder/

3. exp major depression/

4. exp dysthymia/

5. exp mood disorder/

6. depress*.ab,ti.

7. major depressive disorder.ab,ti.

8. MDD.ab,ti.

9. dysthymi*.ab,ti.

10. persistent depressive disorder.ab,ti.

11. depressive disorder*.ab,ti.

12. depressive symptom*.ab,ti.

13. depressive episode*.ab,ti.

14. unipolar depression.ab,ti.

15. mood disorder*.ab,ti.

16. affective disorder*.ab,ti.

17. subclinical depression.ab,ti.

18. subthreshold depression.ab,ti.

Block B — Chatbot / Conversational AI (Lines 19–35)

19. chatbot*.ab,ti.

20. chat bot*.ab,ti.

21. conversational agent*.ab,ti.

22. conversational AI.ab,ti.

23. dialogue system*.ab,ti.

24. dialog system*.ab,ti.

25. virtual therapist*.ab,ti.

26. virtual agent*.ab,ti.

27. relational agent*.ab,ti.

28. embodied agent*.ab,ti.

29. mental health bot*.ab,ti.

30. Woebot.ab,ti.

31. Wysa.ab,ti.

32. Tess.ab,ti.

33. Youper.ab,ti.

34. Replika.ab,ti.

35. Ellie.ab,ti.

Combination

36. 1 OR 2 OR 3 OR 4 OR 5 OR 6 OR 7 OR 8 OR 9 OR 10 OR 11 OR 12 OR 13 OR 14 OR 15 OR 16 OR 17 OR 18

37. 19 OR 20 OR 21 OR 22 OR 23 OR 24 OR 25 OR 26 OR 27 OR 28 OR 29 OR 30 OR 31 OR 32 OR 33 OR 34 OR 35

38. 36 AND 37

1. **Embase (694)**

(

exp depressive disorder/

OR exp depression/

OR depress*.ti,ab.

OR "major depressive disorder".ti,ab.

OR MDD.ti,ab.

OR dysthymi*.ti,ab.

OR "depressive symptom*".ti,ab.

OR "mood disorder*".ti,ab.

OR "affective disorder*".ti,ab.

)

AND

(

exp chatbot/

OR chatbot*.ti,ab.

OR "AI chatbot*".ti,ab.

OR "conversational agent*".ti,ab.

OR "dialogue system*".ti,ab.

OR "virtual agent*".ti,ab.

OR "virtual therapist*".ti,ab.

OR Woebot.ti,ab.

OR Wysa.ti,ab.

OR Tess.ti,ab.

)

AND

(

exp psychotherapy/

OR exp cognitive behavioral therapy/

OR intervention*.ti,ab.

OR therap*.ti,ab.

OR treatment*.ti,ab.

OR counsel*.ti,ab.

OR psychotherap*.ti,ab.

OR "cognitive behavioral therapy".ti,ab.

OR CBT.ti,ab.

OR "digital mental health".ti,ab.

OR "digital therap*".ti,ab.

OR "psychological intervention*".ti,ab.

)

AND

(

random*.ti,ab.

OR randomized.ti,ab.

OR randomised.ti,ab.

OR RCT.ti,ab.

OR "controlled trial".ti,ab.

)

Block A — Depression (Lines 1–20)

1. exp depressive disorder/

2. exp major depression/

3. exp dysthymic disorder/

4. exp unipolar depression/

5. exp reactive depression/

6. exp endogenous depression/

7. exp mood disorders/

8. depress*.ab,ti.

9. major depressive disorder.ab,ti.

10. MDD.ab,ti.

11. dysthymi*.ab,ti.

12. persistent depressive disorder.ab,ti.

13. depressive disorder*.ab,ti.

14. depressive symptom*.ab,ti.

15. depressive episode*.ab,ti.

16. unipolar depression.ab,ti.

17. mood disorder*.ab,ti.

18. affective disorder*.ab,ti.

19. subclinical depression.ab,ti.

20. subthreshold depression.ab,ti.

Block B — Chatbot / Conversational AI (Lines 21–37)

21. chatbot*.ab,ti.

22. chat bot*.ab,ti.

23. conversational agent*.ab,ti.

24. conversational AI.ab,ti.

25. dialogue system*.ab,ti.

26. dialog system*.ab,ti.

27. virtual therapist*.ab,ti.

28. virtual agent*.ab,ti.

29. relational agent*.ab,ti.

30. embodied agent*.ab,ti.

31. mental health bot*.ab,ti.

32. Woebot.ab,ti.

33. Wysa.ab,ti.

34. Tess.ab,ti.

35. Youper.ab,ti.

36. Replika.ab,ti.

37. Ellie.ab,ti.

Combination

38. 1 OR 2 OR 3 OR 4 OR 5 OR 6 OR 7 OR 8 OR 9 OR 10 OR 11 OR 12 OR 13 OR 14 OR 15 OR 16 OR 17 OR 18 OR 19 OR 20

39. 21 OR 22 OR 23 OR 24 OR 25 OR 26 OR 27 OR 28 OR 29 OR 30 OR 31 OR 32 OR 33 OR 34 OR 35 OR 36 OR 37

40. 38 AND 39
