# Supplementary material for: Therapeutic Interaction Features of AI Chatbots in Depression Interventions: Systematic Review and Meta-Analysis
Source: J Med Internet Res. 2026 Jun 30;28:e88697. doi: 10.2196/88697 (PMC13318397; doi:10.2196/88697)
Supplement: Multimedia Appendix 2 [file jmir-v28-e88697-s002.docx]

**Supplementary material 2**

Six predefined interaction features were assessed: interaction frequency, emotional responsiveness, Self-disclosure encouraged, dialogue depth, feedback strategy, and user agency level. Each feature was rated on a five-point scale (1 = very low, 5 = very high) by two independent reviewers. A third reviewer adjudicated when ratings differed by ≥1 point, and the final score for each feature was calculated as the mean of the available ratings. Inter-rater reliability across all ratings was good (overall ICC = 0.71).

Studies were subsequently categorised into high (≥ 3.75) and low (≤ 3.5) subgroups for the meta-analysis. These thresholds were chosen to distinguish consistently high- versus low-scoring studies, leaving out ambiguous mid-range values.

**Supplementary Table S2. Ratings of chatbot interaction features across included studies**

| **Study ID** | **Interaction frequency** | **Emotionally responsiveness** | **Self-disclosure encouraged** | **Dialogue depth** | **Feedback strategy** | **User agency level** |
| --- | --- | --- | --- | --- | --- | --- |
| Chen et al, 2025 [40] | 3.75 | 3 | 3 | 3 | 3 | 3 |
| Fitzpatrick et al, 2017 [41] | 3.75 | 3.5 | 3.5 | 3 | 4 | 3 |
| He et al, 2022 [42] | 4 | 4.75 | 4 | 3.75 | 4 | 3.75 |
| Kang and Hong, 2024 [43] | 3 | 3.75 | 3 | 3 | 3 | 3 |
| Karkosz et al, 2024 [44] | 3 | 3.5 | 3.25 | 3 | 3 | 3.5 |
| Liu et al, 2022 [45] | 3 | 4.5 | 3 | 3.5 | 3 | 4.25 |
| Sabour et al, 2023 [44] | 3 | 2.75 | 3 | 3.75 | 3 | 3 |
| Tong et al, 2024 [45] | 3 | 3 | 3.75 | 3 | 3.25 | 3 |
| Ulrich et al, 2024 [46] | 3 | 3 | 2.5 | 2 | 3 | 4 |
| Vereschagin et al, 2024 [47] | 3 | 3 | 2.75 | 2.5 | 3 | 4 |
| Yasukawa et al, 2024 [48] | 4 | 3 | 2.75 | 3 | 3 | 3 |

References:

40. Chen C, Lam KT, Yip KM, et al. Comparison of an AI chatbot with a nurse hotline in reducing anxiety and depression levels in the general population: pilot randomized controlled trial. JMIR Hum Factors. Mar 6, 2025;12:e65785. [doi: ] [Medline: 40048637]

41. Fitzpatrick KK, Darcy A, Vierhile M. Delivering cognitive behavior therapy to young adults with symptoms of depression and anxiety using a fully automated conversational agent (Woebot): a randomized controlled trial. JMIR Ment Health. Jun 6, 2017;4(2):e19. [doi: ] [Medline: 28588005]

42. He Y, Yang L, Zhu X, et al. Mental health chatbot for young adults with depressive symptoms during the COVID-19 pandemic: single-blind, three-arm randomized controlled trial. J Med Internet Res. Nov 21, 2022;24(11):e40719. [doi: ] [Medline: 36355633]

43. Kang B, Hong M. Digital interventions for reducing loneliness and depression in Korean college students: mixed methods evaluation. JMIR Form Res. Sep 12, 2024;8:e58791. [doi: ] [Medline: 39264705]

44. Karkosz S, Szymański R, Sanna K, Michałowski J. Effectiveness of a web-based and mobile therapy chatbot on anxiety and depressive symptoms in subclinical young adults: randomized controlled trial. JMIR Form Res. Mar 20, 2024;8(1):e47960. [doi: ] [Medline: 38506892]

45. Liu H, Peng H, Song X, Xu C, Zhang M. Using AI chatbots to provide self-help depression interventions for university students: a randomized trial of effectiveness. Internet Interv. Mar 2022;27:100495. [doi: ] [Medline: 35059305]

46. Sabour S, Zhang W, Xiao X, et al. A chatbot for mental health support: exploring the impact of Emohaa on reducing mental distress in China. Front Digit Health. 2023;5:1133987. [doi: ] [Medline: 37214342]

47. Tong ACY, Wong KTY, Chung WWT, Mak WWS. Effectiveness of topic-based chatbots on mental health self-care and mental well-being: randomized controlled trial. J Med Internet Res. Apr 30, 2025;27:e70436. [doi: ] [Medline: 40306635]

48. Ulrich S, Lienhard N, Künzli H, Kowatsch T. A chatbot-delivered stress management coaching for students (MISHA App): pilot randomized controlled trial. JMIR Mhealth Uhealth. Jun 26, 2024;12:e54945. [doi: ] [Medline: 38922677]

49. Vereschagin M, Wang AY, Richardson CG, et al. Effectiveness of the Minder mobile mental health and substance use intervention for university students: randomized controlled trial. J Med Internet Res. Mar 27, 2024;26:e54287. [doi: ] [Medline: 38536225]

50. Yasukawa S, Tanaka T, Yamane K, et al. A chatbot to improve adherence to internet-based cognitive-behavioural therapy among workers with subthreshold depression: a randomised controlled trial. BMJ Ment Health. Jan 10, 2024;27(1):e300881. [doi: ] [Medline: 38199786]
