# Supplementary material for: Therapeutic Interaction Features of AI Chatbots in Depression Interventions: Systematic Review and Meta-Analysis
Source: J Med Internet Res. 2026 Jun 30;28:e88697. doi: 10.2196/88697 (PMC13318397; doi:10.2196/88697)
Supplement: Multimedia Appendix 3 [file jmir-v28-e88697-s003.docx]

**Supplementary material 3**

To further assess the robustness of the pooled estimates, a leave-one-out sensitivity analysis was conducted by sequentially excluding each included study. The results showed that the direction of the pooled effect remained consistent across all iterations, while heterogeneity was substantially reduced only when Fitzpatrick et al [41] was excluded. Detailed results are provided in TableS3.1 and FigureS3.1 - 3.11.

**TableS3.1** Leave-one-out sensitivity analysis of between-study heterogeneity (I²)

| Study ID | I^2^ |
| --- | --- |
| Total | 87% |
| Without Chen et al (2025) [40] | 89% |
| Without Fitzpatrick et al (2017) [41] | 60% |
| Without He et al (2022) [42] | 88% |
| Without Kang and Hong (2024) [43] | 89% |
| Without Karkosz et al (2024) [44] | 89% |
| Without Liu et al (2022) [45] | 87% |
| Without Sabour et al (2023) [46] | 88% |
| Without Tong et al (2024) [47] | 88% |
| Without Ulrich et al (2024) [48] | 88% |
| Without Vereschagin et al (2024) [49] | 87% |
| Without Yasukawa et al (2024) [50] | 88% |


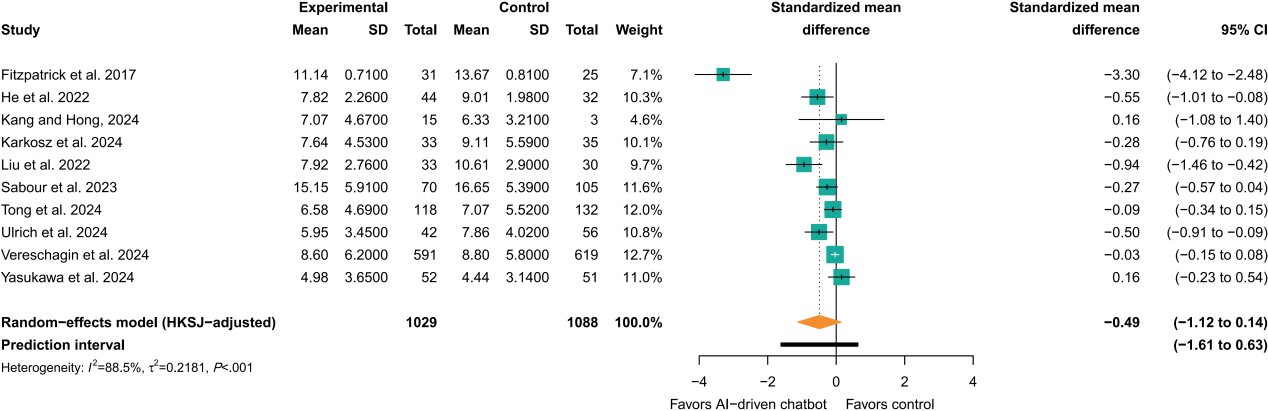


**FigureS3.1** Forest plot of the overall clinical effect of AI-driven chatbots on depressive symptoms. Sensitivity analysis excluding Chen et al [40]. Studies included in the meta-analysis are referenced as [41-50].


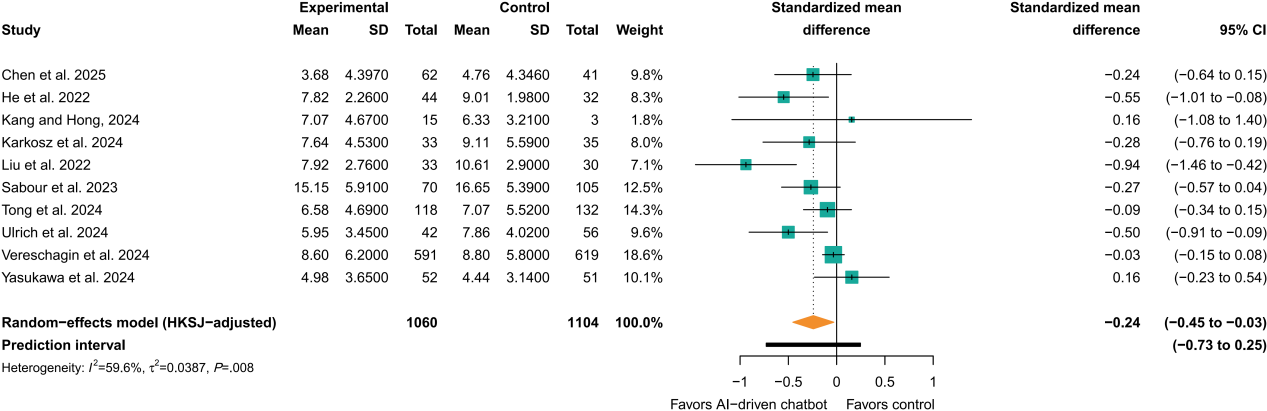


**FigureS3.2** Forest plot of the overall clinical effect of AI-driven chatbots on depressive symptoms. Sensitivity analysis excluding Fitzpatrick et al [41]. Studies included in the meta-analysis are referenced as [40, 42-50].


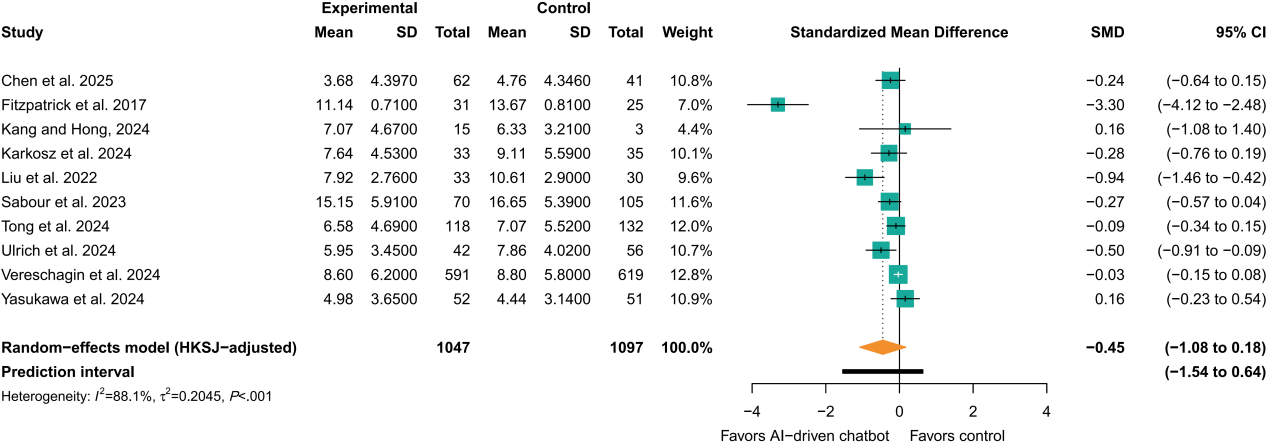


**FigureS3.3** Forest plot of the overall clinical effect of AI-driven chatbots on depressive symptoms. Sensitivity analysis excluding He et al [42]. Studies included in the meta-analysis are referenced as [40, 41 43-50].


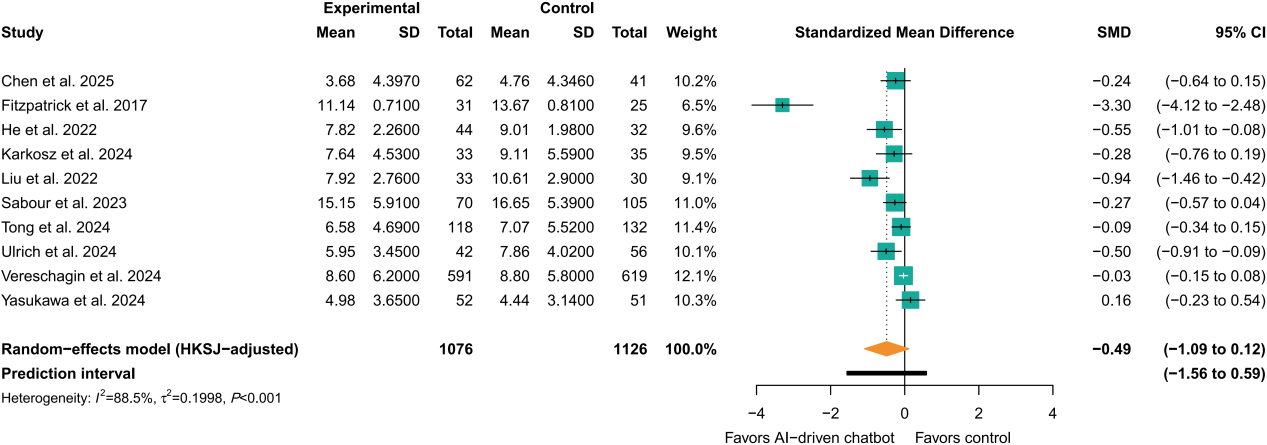


**FigureS3.4** Forest plot of the overall clinical effect of AI-driven chatbots on depressive symptoms. Sensitivity analysis excluding Kang et al [43]. Studies included in the meta-analysis are referenced as [40-42, 44-50].


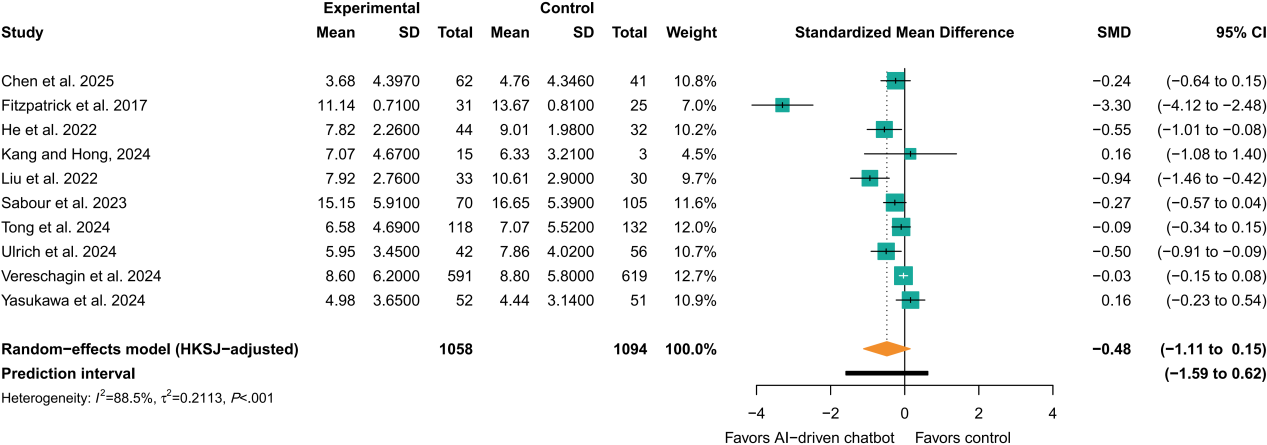


**FigureS3.5** Forest plot of the overall clinical effect of AI-driven chatbots on depressive symptoms. Sensitivity analysis excluding Karkosz et al [44]. Studies included in the meta-analysis are referenced as [40-43, 45-50].

**
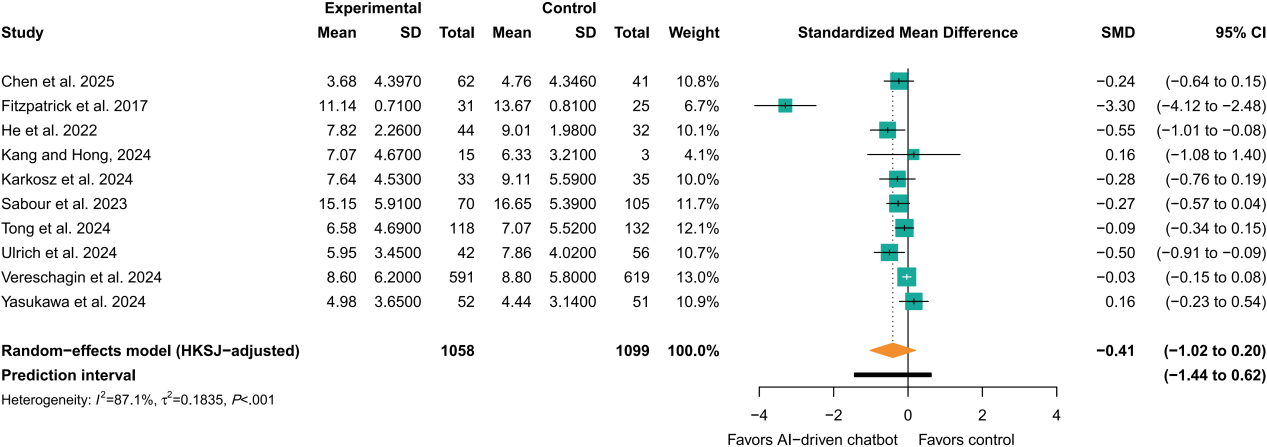
**

**FigureS3.6** Forest plot of the overall clinical effect of AI-driven chatbots on depressive symptoms. Sensitivity analysis excluding Liu et al [45]. Studies included in the meta-analysis are referenced as [40-44, 46-50].


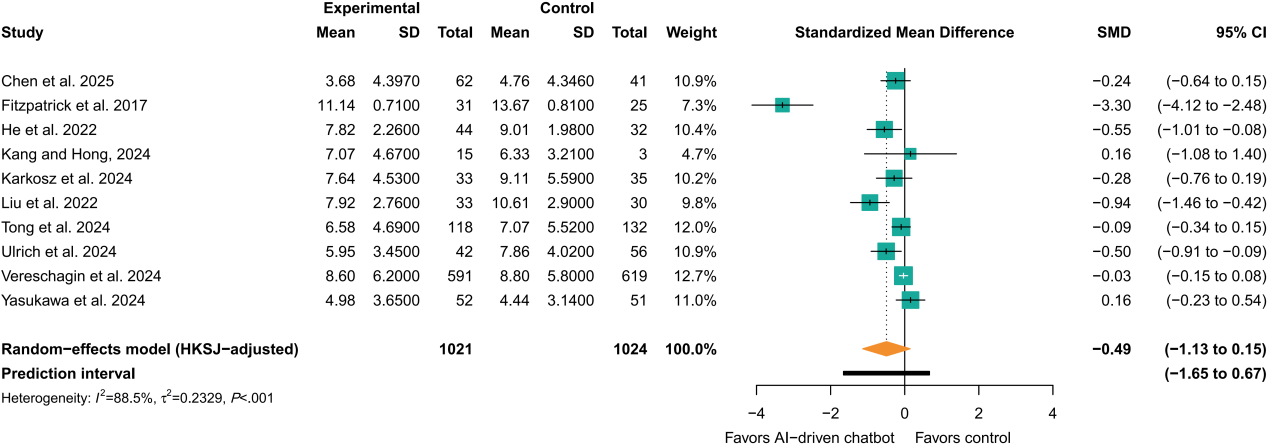


**FigureS3.7** Forest plot of the overall clinical effect of AI-driven chatbots on depressive symptoms. Sensitivity analysis excluding Sabour et al [46]. Studies included in the meta-analysis are referenced as [40-45, 47-50].


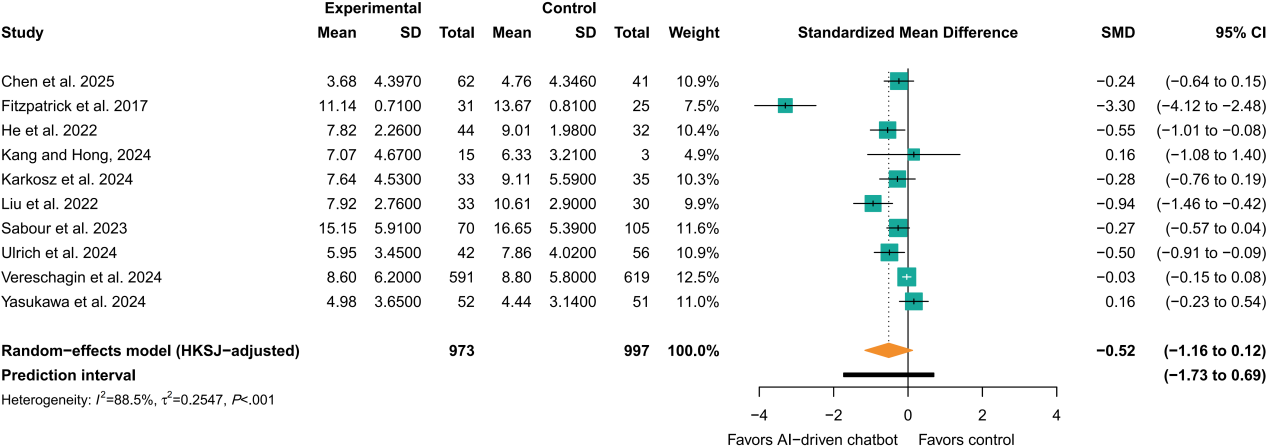


**FigureS3.8** Forest plot of the overall clinical effect of AI-driven chatbots on depressive symptoms. Sensitivity analysis excluding Tong et al [47]. Studies included in the meta-analysis are referenced as [40-46, 48-50].


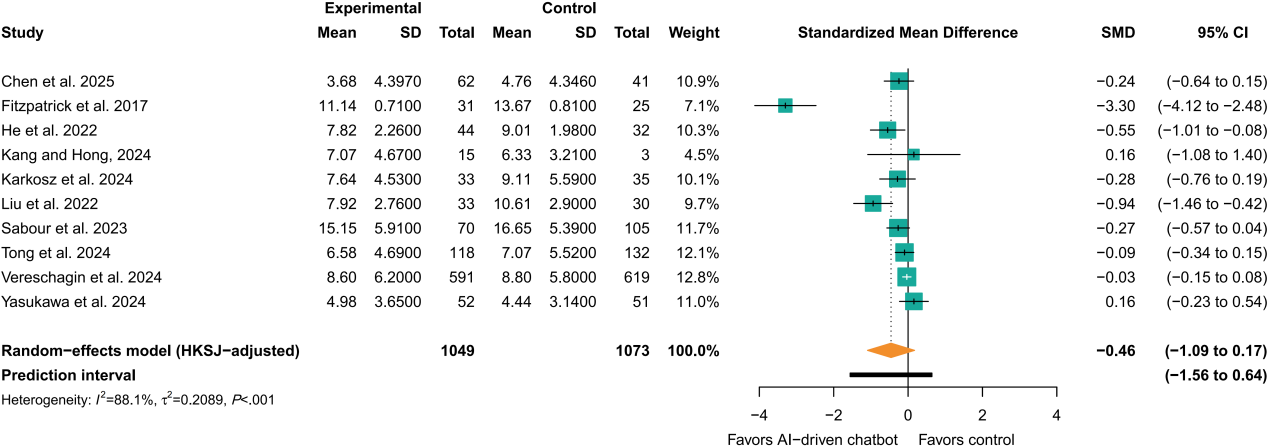


**FigureS3.9** Forest plot of the overall clinical effect of AI-driven chatbots on depressive symptoms. Sensitivity analysis excluding Ulrich et al [48]. Studies included in the meta-analysis are referenced as [40-47, 49,50].


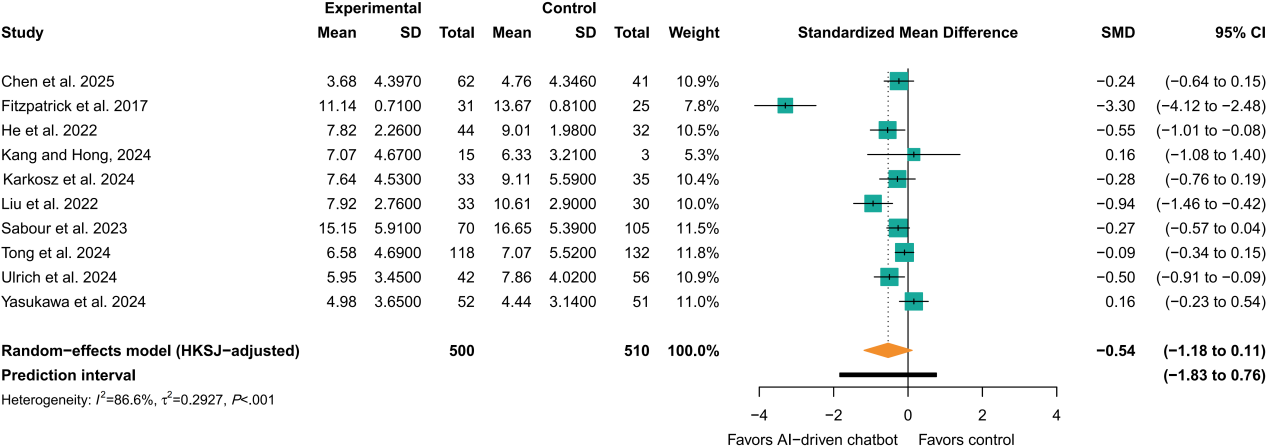


**FigureS3.10** Forest plot of the overall clinical effect of AI-driven chatbots on depressive symptoms. Sensitivity analysis excluding Vereschagin et al [49]. Studies included in the meta-analysis are referenced as [40-48, 50].


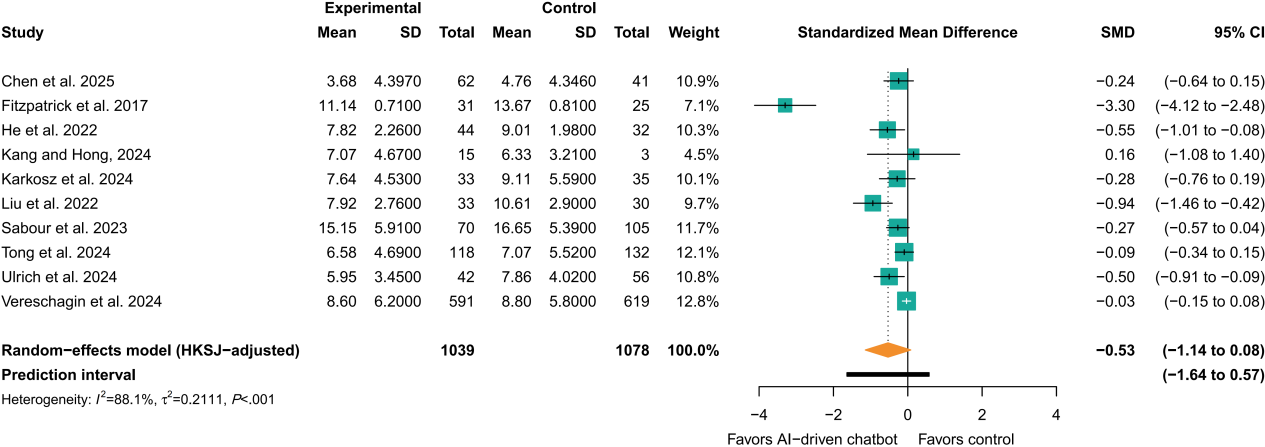


**FigureS3.11** Forest plot of the overall clinical effect of AI-driven chatbots on depressive symptoms. Sensitivity analysis excluding Yasukawa et al [50]. Studies included in the meta-analysis are referenced as [40-49].

References:

40. Chen C, Lam KT, Yip KM, et al. Comparison of an AI chatbot with a nurse hotline in reducing anxiety and depression levels in the general population: pilot randomized controlled trial. JMIR Hum Factors. Mar 6, 2025;12:e65785. [doi: 10.2196/65785] [Medline: 40048637]

41. Fitzpatrick KK, Darcy A, Vierhile M. Delivering cognitive behavior therapy to young adults with symptoms of depression and anxiety using a fully automated conversational agent (Woebot): a randomized controlled trial. JMIR Ment Health. Jun 6, 2017;4(2):e19. [doi: 10.2196/mental.7785] [Medline: 28588005]

42. He Y, Yang L, Zhu X, et al. Mental health chatbot for young adults with depressive symptoms during the COVID-19 pandemic: single-blind, three-arm randomized controlled trial. J Med Internet Res. Nov 21, 2022;24(11):e40719. [doi: 10.2196/40719] [Medline: 36355633]

43. Kang B, Hong M. Digital interventions for reducing loneliness and depression in Korean college students: mixed methods evaluation. JMIR Form Res. Sep 12, 2024;8:e58791. [doi: 10.2196/58791] [Medline: 39264705]

44. Karkosz S, Szymański R, Sanna K, Michałowski J. Effectiveness of a web-based and mobile therapy chatbot on anxiety and depressive symptoms in subclinical young adults: randomized controlled trial. JMIR Form Res. Mar 20, 2024;8(1):e47960. [doi: 10.2196/47960] [Medline: 38506892]

45. Liu H, Peng H, Song X, Xu C, Zhang M. Using AI chatbots to provide self-help depression interventions for university students: a randomized trial of effectiveness. Internet Interv. Mar 2022;27:100495. [doi: 10.1016/j.invent.2022.100495] [Medline: 35059305]

46. Sabour S, Zhang W, Xiao X, et al. A chatbot for mental health support: exploring the impact of Emohaa on reducing mental distress in China. Front Digit Health. 2023;5:1133987. [doi: 10.3389/fdgth.2023.1133987] [Medline: 37214342]

47. Tong ACY, Wong KTY, Chung WWT, Mak WWS. Effectiveness of topic-based chatbots on mental health self-care and mental well-being: randomized controlled trial. J Med Internet Res. Apr 30, 2025;27:e70436. [doi: 10.2196/70436] [Medline: 40306635]

48. Ulrich S, Lienhard N, Künzli H, Kowatsch T. A chatbot-delivered stress management coaching for students (MISHA App): pilot randomized controlled trial. JMIR Mhealth Uhealth. Jun 26, 2024;12:e54945. [doi: 10.2196/54945] [Medline: 38922677]

49. Vereschagin M, Wang AY, Richardson CG, et al. Effectiveness of the Minder mobile mental health and substance use intervention for university students: randomized controlled trial. J Med Internet Res. Mar 27, 2024;26:e54287. [doi: 10.2196/54287] [Medline: 38536225]

50. Yasukawa S, Tanaka T, Yamane K, et al. A chatbot to improve adherence to internet-based cognitive-behavioural therapy among workers with subthreshold depression: a randomised controlled trial. BMJ Ment Health. Jan 10, 2024;27(1):e300881. [doi: 10.1136/bmjment-2023-300881] [Medline: 38199786]
