# Supplementary material for: Therapeutic Interaction Features of AI Chatbots in Depression Interventions: Systematic Review and Meta-Analysis
Source: J Med Internet Res. 2026 Jun 30;28:e88697. doi: 10.2196/88697 (PMC13318397; doi:10.2196/88697)
Supplement: Multimedia Appendix 5 [file jmir-v28-e88697-s005.docx]

**Supplementary Material 5**

**Table S5.1 GRADE Summary of findings for ai-driven chatbot interventions for depression**

| **Outcome** | **No. of studies (participants)** | **Study design** | **Risk of bias** | **Inconsistency** | **Indirectness** | **Imprecision** | **Publication bias** | **Overall certainty** |
| --- | --- | --- | --- | --- | --- | --- | --- | --- |
| Clinical effectiveness (depressive symptom reduction) | 11 RCTs (n = 2220) | Randomized controlled trials | Not serious (7 low risk; 4 some concerns) | **Very serious** (I² = 87%, substantial heterogeneity across trials) | Not serious (population, intervention, and outcome directly aligned with review question) | Not serious (95% CI −0.76 to −0.16; does not cross null; adequate total sample size) | Not detected (no clear evidence of small study effects) | ⨁⨁◯◯ Low |
| User adherence | 11 RCTs (n = 2762) | Randomized controlled trials | Not serious (7 low risk; 4 some concerns) | **Serious** (I² = 75%, considerable heterogeneity) | Not serious (intervention and adherence definitions consistent with review objective) | **Serious** (95% CI 0.74 to 2.03; crosses line of no effect; wide interval) | Not detected (insufficient evidence of publication bias) | ⨁⨁◯◯ Low |
