# Supplementary material for: Therapeutic Interaction Features of AI Chatbots in Depression Interventions: Systematic Review and Meta-Analysis
Source: J Med Internet Res. 2026 Jun 30;28:e88697. doi: 10.2196/88697 (PMC13318397; doi:10.2196/88697)
Supplement: Multimedia Appendix 6 [file jmir-v28-e88697-s006.docx]

**Supplementary Material 6**

**Table S6 Sensitivity analyses using the Hartung-Knapp-Sidik-Jonkman adjustment**

| **Outcome** | **Subgroup** | **Effect Size** | ***95% CI*** | ***P*** |
| --- | --- | --- | --- | --- |
| Clinical effectiveness | Overall | SMD = -0.46 | -1.02 to 0.10 | .096 |
| Clinical effectiveness | LLM-based | SMD = -0.26 | -0.39 to -0.13 | .004 |
| Clinical effectiveness | Rule-based | SMD = -0.51 | -1.80 to 0.77 | .41 |
| Clinical effectiveness | NLP/ML-based | SMD = -0.58 | -1.38 to 0.22 | .15 |
| User adherence | Overall | OR = 1.22 | 0.57 to 2.62 | .57 |
| User adherence | LLM-based | OR = 5.12 | <0.01 to 8.49e+13 | .62 |
| User adherence | Rule-based | OR = 0.95 | 0.36 to 2.51 | .92 |
| User adherence | NLP/ML-based | OR = 1.87 | 0.22 to 16.28 | .57 |

Hartung-Knapp-Sidik-Jonkman adjustment was applied to all random-effects models. Confidence intervals are wider compared with DerSimonian-Laird estimates, particularly in small subgroups with high heterogeneity.
