# Supplementary material for: Therapeutic Interaction Features of AI Chatbots in Depression Interventions: Systematic Review and Meta-Analysis
Source: J Med Internet Res. 2026 Jun 30;28:e88697. doi: 10.2196/88697 (PMC13318397; doi:10.2196/88697)
Supplement: Multimedia Appendix 7 [file jmir-v28-e88697-s007.docx]

**Supplementary Material 7 funnel plot**

| 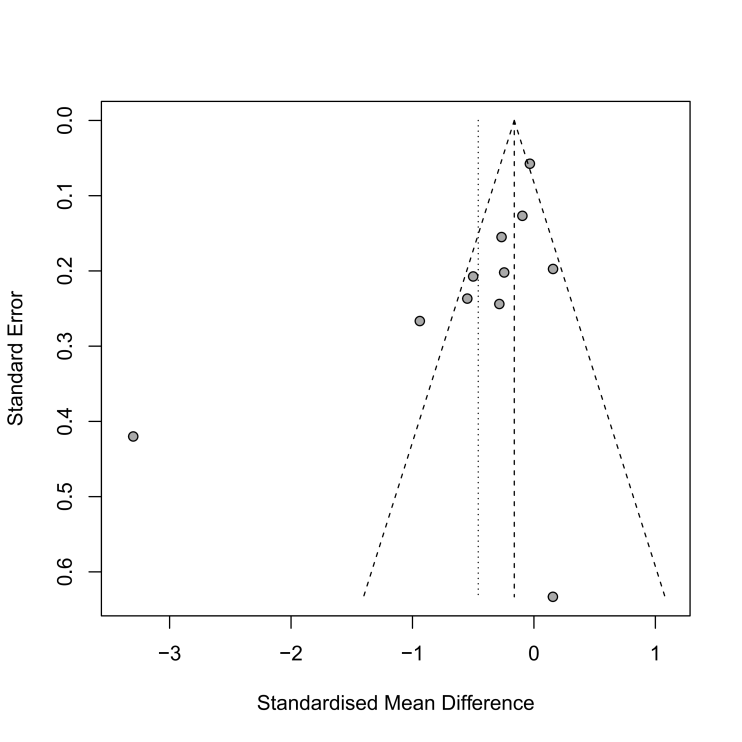 | 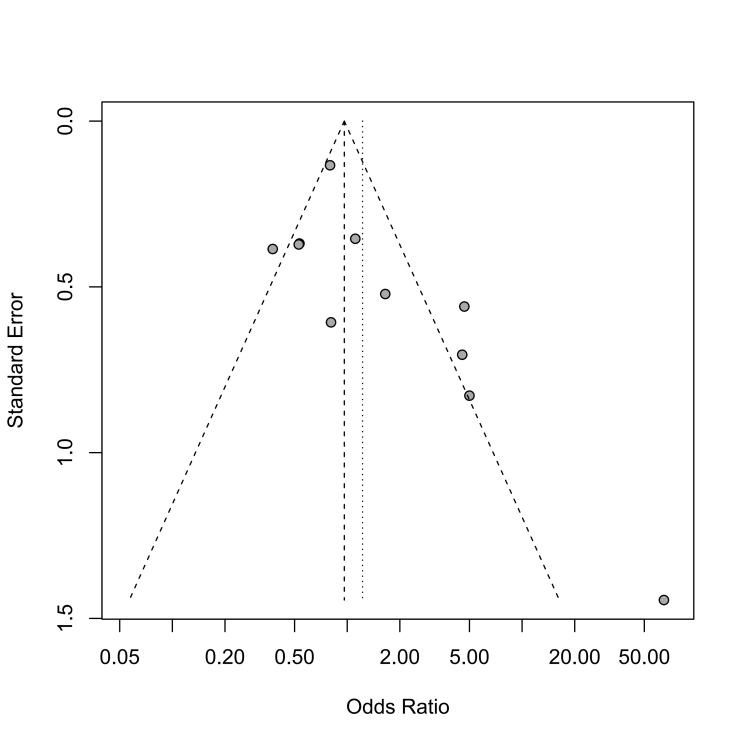 |
| --- | --- |
| Figure S7.1 Funnel plot for clinical effectiveness. | Figure S7.2 Funnel plot for user adherence. |
