# Supplementary material for: Therapeutic Interaction Features of AI Chatbots in Depression Interventions: Systematic Review and Meta-Analysis
Source: J Med Internet Res. 2026 Jun 30;28:e88697. doi: 10.2196/88697 (PMC13318397; doi:10.2196/88697)
Supplement: Checklist 1 [file jmir-v28-e88697-s008.docx]

**PRISMA-S Checklist**

| **Section/topic** | **#** | **Checklist item** | **Location(s) Reported** |
| --- | --- | --- | --- |
| **INFORMATION SOURCES AND METHODS** | | | |
| Database name | 1 | Name each individual database searched, stating the platform for each. | 2.1 Search strategy |
| Multi-database searching | 2 | If databases were searched simultaneously on a single platform, state the name of the platform, listing all of the databases searched. | Not applicable. Databases were searched individually. |
| Study registries | 3 | List any study registries searched. | 2.1 Search strategy: Scopus, Web of Science, PubMed, IEEE Xplore, APA PsycINFO (Ovid), and Embase (Ovid) |
| Online resources and browsing | 4 | Describe any online or print source purposefully searched or browsed (e.g., tables of contents, print conference proceedings, web sites), and how this was done. | Not applicable. No online or print sources were purposefully searched or browsed outside the bibliographic databases. |
| Citation searching | 5 | Indicate whether cited references or citing references were examined, and describe any methods used for locating cited/citing references (e.g., browsing reference lists, using a citation index, setting up email alerts for references citing included studies). | Citation searching was not performed. No cited references or citing references were examined beyond the database searches. |
| Contacts | 6 | Indicate whether additional studies or data were sought by contacting authors, experts, manufacturers, or others. | No additional studies or data were sought by contacting authors, experts, manufacturers, or other stakeholders during the review process. |
| Other methods | 7 | Describe any additional information sources or search methods used. | No additional information sources or search methods beyond the database searches were used in this review. |
| **SEARCH STRATEGIES** | | | |
| Full search strategies | 8 | Include the search strategies for each database and information source, copied and pasted exactly as run. | Full database-specific search strategies for all databases are provided in Supplementary Material 6, copied exactly as executed. |
| Limits and restrictions | 9 | Specify that no limits were used, or describe any limits or restrictions applied to a search (e.g., date or time period, language, study design) and provide justification for their use. | 2.1 Search strategy |
| Search filters | 10 | Indicate whether published search filters were used (as originally designed or modified), and if so, cite the filter(s) used. | No published search filters were used. The search strategies were developed specifically for this review. |
| Prior work | 11 | Indicate when search strategies from other literature reviews were adapted or reused for a substantive part or all of the search, citing the previous review(s). | The search strategies were developed specifically for this review and were not adapted or reused from previously published literature reviews. |
| Updates | 12 | Report the methods used to update the search(es) (e.g., rerunning searches, email alerts). | The database searches were rerun during the revision process to identify any additional studies published after the initial search period. The updated searches were conducted on February 16, 2026. See section 2.1 Search strategy. |
| Dates of searches | 13 | For each search strategy, provide the date when the last search occurred. | The final database searches across all databases were conducted on February 16, 2026. See section 2.1 Search strategy. |
| **PEER REVIEW** | | | |
| Peer review | 14 | Describe any search peer review process. | The search strategy was developed collaboratively by the research team but did not undergo a formal peer review process. |
| **MANAGING RECORDS** | | | |
| Total Records | 15 | Document the total number of records identified from each database and other information sources. | A total of 982 records were identified from database searches: Web of Science (n=461), Scopus (n=163), IEEE Xplore (n=20), PubMed (n=111), APA PsycINFO (n=37), and Embase (n=190). |
| Deduplication | 16 | Describe the processes and any software used to deduplicate records from multiple database searches and other information sources. | Records retrieved from different databases were imported into EndNote for deduplication. Automatic duplicate removal was followed by manual checking to ensure accuracy. |
|  |  |  |  |
| PRISMA-S: An Extension to the PRISMA Statement for Reporting Literature Searches in Systematic Reviews | | |  |
| Rethlefsen ML, Kirtley S, Waffenschmidt S, Ayala AP, Moher D, Page MJ, Koffel JB, PRISMA-S Group. | | |  |
| Last updated February 27, 2020. | |  |  |
